# Spirit of the Foreign Periodicals, &c.

**Published:** 1843-07-01

**Authors:** 


### Spirit of the Foreign Periodicals.

### Hydropathic Tour from Strasbourg to Graefenberg.

We have been not a little amused with a report—it is not distinctly stated whether it be *official* or not—sent by Dr. *Scoutetten*, professor of military surgery at Strasbourg, to the Minister of War, of his tour through the German States to the head quarters of Hydropathy or Hydrotherapeia (as he calls it), with the view of ascertaining its merits and determining the propriety of introducing it into France. The letter is dated the 20th of November, and addressed to "Monsieur le Marechal;" he having graciously favoured the traveller with introductions to the diplomatic agents of the government in the different countries through which he passed. The present remarks, we are more than once somewhat ostentatiously told, are only introductory to a larger and more scientific (!) work on the subject, which will be published forthwith.

Leaving Strasbourg on the 20th of September, I travelled, says M. *Scoutetten*, through the Grand Duchy of Baden, which has only one, and that very small, hydropathic establishment to boast of: it is situated at the foot of the mountains in the Black Forest. The kingdom of Wurtemburg, however, has three; by far the best one is near the small village of Eslingen, in a charming and most healthful district.

The speculators (actionnaires) seem to be vastly well satisfied with the result of their undertaking, as I was told that they are ready to lay out a hundred thousand francs, and more, in the erection of new buildings. The physicians of Stuttgard (the capital) have not taken any interest in the matter, and the system does not seem as yet to have had any influence on the social habits of the community. Not so with Bavaria, which, for the last six or eight years, has been "seriously occupied" with hydrotherapeia.

In 1837, the king sent two of his physicians to Graefenberg, to ascertain the merits of the system; and, since that time, numerous establishments have been set a going in different parts of his kingdom. The most complete one is at Alexandersbad.

Many of the most distinguished physicians of Munich frankly acknowledge that the hydriatic plan, "convenablement appliqué," is a resource of potent efficacy in various diseases which have resisted the ordinary methods of treatment. However this may be, there has, unquestionably, been a marked change in the habits of many persons in the higher ranks of society, since the hydropathic regime has come into fashion. The members of the court have already renounced the use of wine and other exciting beverages; and every one uses the cold bath in the morning. Even the queen and her children "ne se reculent pas" from these refrigerant ablutions. (How interesting!)

What deserves especial notice is that, in the Military Hospital a few miles from Munich, Dr. *Gleich* has become so smitten with admiration of the new system, that he treats every case, whether of internal or of external disease, by it; (we wonder if this be with the sanction of the medical board at head-quarters); and his success has been, he says—as a matter of course—truly surprising. But then M. *Scoutetten* found, by comparing the mortality in this hospital with that in others where the hydropathic treatment was unknown, that in truth there was very little difference to be discovered between them. He continues: "Having traversed Bavaria, I then entered the Austrian dominions, of which Silesia—where the pretty village or hamlet of Graefenberg is situated—forms a part.

"The enthusiasm in favour of the new system throughout Austria is so great with multitudes, that I was credibly informed by the leading banker in Vienna, that many of the *restaurateurs* there, finding that there was no longer any sale for their wine and liqueurs, bethought themselves of retailing water at so much a glass. The water in Vienna itself being rather indifferent, they brought a better kind from Schonbrun.* The use of cold ablutions is now so very general among all classes, that quite a new trade has sprung up in the place; viz. the manufactory and sale of baths of all sorts.

"With the exception, however, of the old Baron *Turckheim* (who, we suppose, is in his dotage), none of the medical men in Vienna have embraced the hydropathic system. Dr. *Guntner*, the physician in ordinary to the Emperor, told me that, while he has witnessed useful results from it in certain instances, he has known others in which most injurious consequences were produced.

"*Priessnitz*, however, has had the good luck of having the royal permission to treat all sorts of diseases on his own plan, and to act in every respect as a licensed physician, although he has never studied in any way the principles of medicine. There are not fewer than six establishments in the neighbourhood of the Austrian capital; but only two of these enjoy much repute. In one of the military hospitals, that at Muhlau, near Inspruck, in the Tyrol, the system has been introduced for the treatment of those diseases which may be considered by the medical officer as suitable for its employment."†

M. *Scoutetten* now proceeded to Graefenberg, which is situated about a hundred leagues to the north of Vienna. It is a mere hamlet, consisting of thirty houses or so on the east slope of a mountain, which rises above the little village of Freywaldau. The air of the place is pure and bracing, and the water is excellent. Here it was that Hydropathy took its rise; and certainly the change, that has been wrought in the district within the space of a very few years, is truly marvellous. The peasants of this part of the country had long been in the habit of using water freely in the treatment of most of their diseases. *Priessnitz*, however, was the first to follow out a regular system. Being more intelligent and inquiring than his fellows, he observed that the benefit obtained was almost always proportionate to the amount of perspiration induced; and having had the misfortune (or we may rather say, the good fortune) to break a couple of his ribs, he undertook to cure himself on the cold water system, and, as a matter of course, succeeded most admirably.

This cure made a great stir in the place, and brought many of the people in the neighbourhood to consult him about their maladies. His fame soon began to spread, and was not long of passing the snowy mountains of Silesia to different parts of Germany.

In 1829, forty-five patients came from remote districts to consult the Graefen-

---

* In *Dresden*, and several other continental towns there have been established regular associations of *hydrophilists*, who regularly correspond and encourage each other in promoting the use of the water regime. These societies, says our author, have a good deal of analogy with the temperance clubs of England and America. If this be the only end and aim of hydropathy, we wish it all possible success.

† "There are now hydropathic establishments, not only in all the leading towns of Germany, but also in Petersburg, in Riga, and in different parts of Belgium and England—one, near London, has been organised on a very large scale by wealthy 'actionnaires.'" What is brother Jonathan about, that he has allowed all other nations to go so much *ahead* of him in this affair? Is it that he still prefers his mint-julep, sangaree, brandy-flip, &c. to the pure element?

berg doctor; in 1832, not fewer than 118; in 1836, 469; and, in 1840, upwards of 1,500.

"In the present day," says our author, "Graefenberg has become the hospital of the incurables of all Europe. I there saw patients from Petersburg, from Moscow and Paris, from London and Philadelphia, from Astracan and Constantinople, and, as a matter of course, from Vienna, Berlin, and several cities of Italy.

"During the present year, the Prince of Nassau, Prince Lichtenstein, the sister of the King of Prussia, the Princess Sapieha, Princess Gortshakoff, the son of the Duke of Sussex, from England, many of the magnates of Hungary, besides a host of countesses and baronesses 'de tout age et de tout pays' have been there.

"What makes all this the more wonderful is, that there is surely nothing in the mode of treatment pursued there that is at all likely to attract the fashionable idler, as is the case at most of the German watering-places. Every thing is conducted in a serious, nay ascetic, manner; the life led is most simple; and the amusements are very few indeed. It was certainly an amusing sight to see so many distinguished personages, accustomed themselves to command, obey with the most religious scrupulousness the prescriptions of a peasant, who can scarcely write his own name. But, although illiterate, *Priessnitz* has received from heaven (!) a rare sagacity, united with great firmness of purpose, and no common strength of judgment. Had he not been so endued, he never could have acquired so wide-spread a celebrity, or been able to carry through his projects with such astonishing success, as he has done. He says little, and never gives any reason for his prescriptions. He takes no note of his cases; but his memory must be excellent, as we have been told that, if five hundred patients are assembled together, he quite recollects every thing he has ordered to each individual. He has already amassed a large fortune, and has purchased a beautiful estate in the neighbourhood of the scene of his exploits."

It has been unjustly said that *Priessnitz* applies the same method of treatment to all his patients alike. This is a mistake. The use indeed of water, in some form or another, is the basis of his treatment on all occasions; but the mode of using the remedy, and the extent to which it is carried, are varied according to the circumstances of each case. The object seems to be to wash away every impurity from the great surfaces of the body, the skin and alimentary passage, and to cause these parts, so to speak, to wash themselves by copious transpiration.

All sorts of baths are used, from the simple foot to the general douche or plunge bath; the temperature of the water varying from 40° to 70° or even 80°, in some cases, when the constitution of the patient is weak and easily affected. Then the fair element is not only drunk profusely, but is also administered more or less frequently in the form of *lavements*. By-the-bye, this French word very happily expresses the meaning of the system. We should not be surprised if, after this, our neighbours claim the discovery of Hydropathy.

It is not necessary to describe particulars:—swathing the body in two or three blankets; the drinking several tumblerfuls of cold water when the skin begins to perspire; the plunging into the cold bath, when every pore is sweating profusely; the subsequent drying, rubbing, and dressing; the walk for an hour, during which six or eight tumblerfuls more of water are drunk; the simple breakfast, at eight o'clock, of milk and plain bread; the second walk for another hour; the undressing and the investiture with the wet sheet at eleven, and the subsequent currying both before and behind; the dressing again and the third walk; the dinner at one o'clock; the fourth walk; the douche bath between three and four o'clock; the fifth walk, &c. &c.

M. *Scoutetten* seems to have been quite pleased with the animating sight of the dinner table, at which not fewer than five hundred persons can be accommodated. There is no dainty put down; nothing but plain joints of meat, vegetables, fruit, and water in abundance: 'voila tout le diner.' And yet it would astonish the pampered inhabitants of most towns to see with what vigorous appetites the *invalids* set to their repast, and what quantities of vivers most of them consume.

By-the-bye, *Priessnitz* seems to treat his patients better than mine host in this country usually does mail-coach travellers; they are not obliged to bolt their food and be off; plenty of time is granted; the dinner being served up 'avec une lenteur Germanique desesperante,' and occupying usually a full hour and a half.

At seven o'clock—the patients having previously had their post-prandial douche-bath, their rubbing down, and their walk 'a grands pas'—the supper evening repast of cold milk and plain household bread is served up.

Each day the patient goes through the same routine of wrapping, sweating, bathing, rubbing, walking, eating, and sleeping.

> "On *cold-served* repetitions he subsists,
> No maiden relishes, no unbroached delights!"

The circle of continually-returning occupations however, Dr. S. says, so completely fills up every hour of the day, that the patients rarely experience any *ennui*. Really if the "whole duty of man" consisted in purging away the impurities of his sinful body by ablutions, no system could be altogether more admirable than that of hydropathy; and *Priessnitz* should be forthwith canonized. At all events, we must all surely admit that his plan is a thousand times better than that of the saints of old, who used to wear their sackcloth shirts unchanged for six months, until they were actually quick with vermin.

No medical man will be likely to dispute the advantages of such a regime as that described above in a variety or chronic ailments, provided there be no coexistent internal diseases of consequence. But what will our readers say when we tell them that Dr. *Scoutetten*, first professor at the military hospital in Strasbourg, actually tells us that the hydropathic mode of treatment is very successful even in the most grave acute diseases, especially in typhus fever and in obstinate dysenteries. *Priessnitz* himself and his German disciples are much more prudent; for they receive none but chronic cases—crafty rogues!

Some reader may here very naturally ask, how long is the washing and scrubbing system to be continued in the average number of cases? Dr. S. replies; from one, to six, twelve, or twenty months. He saw, indeed, some patients at Graefenberg who had been there for two and three years: the Prince Lichtenstein has not left it for four years. (Query. Has this worthy potentate any thing to do? or is it that his purse is not very heavily laden?) The following bit of information as to what classes of diseases are most benefited by the water-cure, we must give in the doctor's own words:—

"I should say that success is almost certain in gout, rheumatism, all abdominal affections, (!) scrofula, and inveterate syphilis; that it is less uniform in cutaneous diseases, in recent syphilis, in paralysis, and other affections of the nervous system; and lastly, that the treatment generally fails in chronic affections of the chest!!"

After this scientific general announcement, the Doctor gives reports of several cases of wonderful cures which he saw at Graefenberg: they all occurred, it would seem, among the big folks of the earth.

An old French General, 70 years of age—who had a liver that extended over half his belly, and who, after consulting all the doctors of any repute in Paris, Vienna, and Italy, had actually, on one occasion, gone off to the Caucasus to drink hot water there—was, at length, induced to put himself under *Priessnitz's* regime, when every other means had failed. His liver, we are told, is now reduced to a 'juste-milieu' dimensions, and he has quite recovered his health and strength!

The case of Dr. *Bulard* is still more extraordinary. His liver had actually become so enormously large, as to make his abdomen as big as that of a woman nine months gone with child. He had been four months at Graefenberg, when seen by Dr. *Scoutetten*, who "feels confident that, by persevering in the treatment, he will be perfectly cured!"*

The son of Prince S———, 12 years of age, had for several years been subject to frequent returns of unappeasable vomiting, which usually lasted for one or two weeks at a time, and which had resisted every remedy that had been tried. He was speedily cured!

The Countess P——, 65 years old, had for many years suffered (poor soul,) with excessive great pain and stiffness in her hands and feet. When she came to Graefenberg, the joints seemed to be almost anchylosed. Under the hydriatic regime she has again become plump and active, can walk famously, eats like a trooper, and writes with ease; nay, she even "touchait le piano devant moi," says the doctor!!

*Priessnitz* is a lucky fellow; he not only gets lots of cash from his patients, but has monuments raised by them in his honour. A full-sized metal statue of a lion—by-the-bye, our own Hygeist, *Morrison*, used to sport one on the top of his house in the New Road here—faces the village of Freywaldau, with commemorative inscriptions on its pedestal; and on the road to Graefenberg, stands a monumental fountain, surmounted with the figure of a star, emblematic of the 'avenir' of hydrotherapeia.

Dr. *Scoutetten*, after lauding in the highest terms the success of *Priessnitz*'s practice, acknowledges that he (the latter) commits some egregious mistakes in diagnosis; he cannot distinguish between nervous and aneurismatic affections of the heart, or between simple gonorrhœa and the urethral discharges connected with the presence of stricture. But we much doubt whether the peasant of Silesia has ever been guilty of such digraceful malpraxis as the professor of Strasbourg.

What are we to think of a regularly-educated medical man trying his experiments of hydropathic treatment on a man in the last stage of typhus fever, "dont l'existence ne se revelait que par un bruissement à la region du coeur?" Had he told us nothing else of his practice but this, we should have had quite enough to know what amount of faith we should give to his opinion on any medical subject.

We cannot dismiss the account of this professor's hydropathic trip without expressing our astonishment at the many proofs which it exhibits of his ignorant credulity. Is it that he has fallen into his dotage, and cannot distinguish between the random successes of a clever empiricism and the well-calculated results of legitimate practice? or is he influenced by some mercenary motives, and supposes that he may 'make a good thing,' like several of our own countrymen, of the new system?

To do M. *Scoutetten*, however, no injustice, we may here mention that the water-cure has recently been tried in one of the hospitals of Paris, and that a full report of the practice has been duly sent to the Council of Health. M. *Devergier*, one of the physicians of the Hôpital St. Louis, entrusted several cases of cutaneous disease—psoriasis and lepra—and two of chronic rheumatism, to the care of M. *Wertheim*, to be treated on hydropathic principles. The results on the whole were any thing but very satisfactory; three only of the first set being cured: both rheumatic patients were much benefited.

---

* Most unfortunately for the Doctor's skill in prognosis, his poor *confrere* has recently died at Dresden.

This, however, is certainly not giving a fair trial to the system; for one part only of the *regime* required can be duly carried out in a metropolitan hospital; the bracing air, the mountain walk, &c.&c. are awanting. That not a few cases of chronic disease, if unattended with internal mischief, must be benefited by the hydropathic treatment pursued as *Priessnitz* does it, among the mountains of Silesia, no reasonable medical man will doubt; for, after all, what does this treatment come to?—to very nearly what jockeys and pugilists in this country would call capital *training*. Instead of putting on half-a-dozen of great-coats, and walking briskly for an hour or two, until the skin is streaming with perspiration, the Hydropathist is wrapped up in blankets like a mummy and left to stew for some time in his own liquor, to be then sluiced with cold water and well curried down. The professors of both arts follow nearly the same regulations about diet and exercise.

As to the application of the hydropathic treatment to the treatmeut of inveterate skin diseases, we can see no advantages likely to accrue from it, that might not be much more easily and quite as effectually obtained by a regular system of vapour-baths, in conjunction with the use of such means as must improve the general health, such as wholesome diet, exercise in the open air, &c.

---

### M. CHOMEL ON CLINICAL OBSERVATION.

In his opening lecture at the Faculty of Medicine, this distinguished teacher made some excellent remarks on what he called 'therapeutic experimentation,' and pointed out the rules which must be observed by medical observers, if they hope to arrive at any thing like certainty in their conclusions. After declaring his opinion very decidedly against most of the recent attempts at innovation, of which therapeutics are incessantly the object, he very ably shewed that the primary and essential foundation of all good experimentation is the knowledge of three things—the remedy itself, the patient, and the existing disease.

With respect to the *remedy* to be employed, the medical man should be thoroughly acquainted with its nature and composition, the pharmaceutic preparations of it that are in use, and the adulterations to which these are liable. Without such a knowledge, he will be apt to attribute to one substance the properties that belong to another, and to a compound medicine those of a simple one, &c. For example, it is now well known that the virtues of the syrup of asparagus—so long in favour as a diuretic—are entirely owing to the extract of digitalis introduced into its composition.

It is equally necessary to attend to the age, the temperament, the occupation, &c. of the *patient*, when any therapeutic experiment is made; and with respect to the *disease* itself, the physician must consider its nature, duration, its usual course and natural termination. As a general remark, it may be asserted that a series of experiments will be the more valuable and instructive, in proportion as the existing disease is the more fixed in its seat, and the less complex in its character.

Supposing now that the conditions, of which we have been speaking, are all well determined, the question comes to be, how should we proceed to make trial of any therapeutic means? First of all, it should be tried alone, and not combined with other remedies; else how can we determine what results belong to the operation of the one, and what to that of the other? Secondly, we must be sure that it has been properly administered or used: hence the importance of medical men often administering certain medicines themselves, or, at all events, leaving the most exact directions to the attendants. If we have to do with any very energetic medicine, we should try its effects repeatedly on the lower animals, before resorting to its use with our fellow-creatures. And lastly, we should remember that mere change of place, the vicissitudes of the weather, the influence of mental emotion, the intervention of other diseases or of some other accidental circumstances, may all exercise on the progress of any disease such important modifications as will materially alter the results of any therapeutic experiment.

M. *Chomel* illustrated the truth of these precautions very happily by alluding to what occurred in his own practice very lately. He wished to try the effects of the Humulus lupulus as a remedy in intermittent fevers, and he accordingly determined to give the remedy a fair trial in 22 cases, which were under his care. But it so happened that the trial could not be made for a few days; and, by that time, the fever had spontaneously ceased in no fewer than 19 of the cases: in the remaining three, the powdered hop was given in very large doses, but with no obvious benefit. How erroneous would the conclusion here have been, had the medicine been administered to all the patients as early as it was first intended!—*L'Examinateur Medical.*

*Remarks.*—The observations of a calm observing physician like M. *Chomel* are always valuable, especially when, as in his case, he does not belong to any particular sect or school of medicine. What a contrast between the reflections of the physician of the Hôtel Dieu on any practical subject, and those of the ardent professor of La Charité hospital, M. *Bouillaud!* The one, like a cautious but bold general, makes himself thoroughly acquainted with every thing appertaining to his own army and that of the enemy, the nature of the ground, the character of the troops, the spirit of the officers and so forth, before he comes to blows; the other reminds you of a fiery captain of hussars, that plunges into the midst of the fight, without a moment's consideration whether an equal advantage might not be obtained without the sacrifice of a single life. Be assured there is no little analogy between the duties of the military, and those of the medical, profession. The physician is the general; the pharmacopœia—using this term in a very wide acceptation—is his army; the disease is the enemy opposed to him. He has his skirmishes, as well as his pitched battles, to fight. He has his long campaigns, as well as his sudden encounters, to be prepared for. He has his out-posts to look after, his guards to set, his entrenchments to fortify, his garrisons to subsist. His commissariat, as well as his gunnery and ammunition stores, must be attended to. When apparently most secure, he is liable to sudden surprises and inroads; the work of one hour may upset the exertions of a whole month; the events of one day may ruin the labour of years. And last of all, he must be prepared for many emergencies which no human vigilance can foresee, and no human prudence can prevent. A passing storm may lay prostrate in a moment his wisest preparations; or the undermining influence of fear and despondency may render them all of no avail. No set of men surely ought to know better, or feel more deeply, than the soldier and the physician, the full force of the saying that "the race is not always to the swift, nor the battle to the strong."—*Rev.*

---

### M. Chomel on the Diagnosis of Pneumonia.

In one of his recent clinical lectures at the Hôtel Dieu, M. *Chomel* made the following remarks on the importance of shivering as a diagnostic sign of thoracic inflammation, in commenting upon a case of pneumonia that was in the wards at the time.

"I took much pains in questioning this patient, to ascertain whether she had experienced any chill, before the commencement of the attack; and her reply was always in the negative. This circumstance appears to me of importance; and it is therefore designedly that I now call your attention to the subject, seeing that it is the professed opinion of many physicians that pneumonia, like articular rheumatism, may generally be traced to the influence of damp and cold. The results however of my own experience, as well as of that of many others whom I know, are quite opposed to this opinion. No doubt it often happens that pneumonic patients will be found to have been chilled some time before the attack came on; but assuredly the chill is not the only, nor even the principal, cause of the disease. If we enquire into the particulars of a case, we shall generally find that there was a predisposition to the malady present in the system at the time, and that the chill only accelerated the development of the mischief. It was merely the occasion, so to speak, of the explosion of a pre-existing morbid state; just in the same manner as a simple indigestion may be the exciting cause of a gastric inflammation in a person, in whom there is a strong disposition to this disease.

"But the same remark does not hold true of shivering when this occurs at the commencement of a disease. In my opinion it is an almost invariable sign of pulmonary inflammation. Whenever, therefore, this symptom is or has been present, the physician will do wisely to direct his attention to the chest; and very generally, at least according to my experience, he will find that an inflammatory process has been set up in the lungs—unless indeed some well-marked symptoms clearly point to another organ as the seat of suffering. I do not deny, as a matter of course, that an attack of peritonitis, enteritis, &c., is sometimes ushered in with shivering; all that I mean to assert is, that this symptom is infinitely more common as a precursor of pneumonia than of any other inflammation. Hence in practice, whenever any of my patients has a well-marked shivering fit, even although other symptoms indicative of disease elsewhere be strongly marked, I at once suspect that the lungs are more or less seriously affected. On very many occasions indeed, this symptom alone has sufficed to suggest to me the right diagnosis, while other medical men, who have seen the case at the same time, have formed a very different opinion.

"There is another character which equally deserves the attentive consideration of the physician—and that is the pain in the side. In pleuro-pneumonia the pain is generally seated in the region of the mamma, although the affected part of the lung does not correspond to this point, or perhaps extends much beyond it. It has been suggested, in the way of explanation, that there is a greater degree of friction between the pulmonic and the costal pleuræ at this point than at any other, and that this may be the cause of the phenomenon in question. But if such were the case, the pain should surely not be limited to so circumscribed a spot, but should extend over all the surface where this greater friction is experienced; and we might expect, moreover, that it should change its locality—which certainly does not hold true. No satisfactory explanation has hitherto been offered of this symptom, and we must therefore confess our ignorance upon the point."—*Gazette des Hôpitaux.*

*Remarks.*—M. *Chomel* surely attaches an undue importance to the occurrence of shivering as a distinctive sign of pneumonia: he represents it as almost pathognomonic of the disease. But are not all the Pyrexiæ usually ushered in with this symptom? and however frequent bronchitic and pulmonic inflammation unquestionably is, as a complication of febrile disorders, it cannot be regarded as of almost constant occurrence. No one symptom, however valuable, should ever be trusted to inordinately.

### MM. Andral and Gavarret on the Development of Vegetable Productions in Albuminous Fluids.

In prosecuting their enquiries respecting the changes which the blood undergoes in the course of various diseases, the attention of these gentlemen was drawn to a communication which M. *Liebig* recently addressed to the Academy of Sciences, and in which—after stating that in his opinion fibrine and albumen were substances perfectly identical in their nature, and that he had obtained globules of blood from the former—he said, "I have succeeded in precipitating albumen in the form of globules, by adding a sufficient quantity of water to serum which has been rendered neutral by an acid."

Here was a most important announcement; and the question that immediately suggested itself for the consideration of the physiologist, was whether albumen could, by simply undergoing a change in form, constitute the nuclei of the red globules of the blood.

MM. *Andral* and *Gavarett* set about repeating the experiments of the distinguished German chemist, and they found to their astonishment that the corpuscles—more or less exactly rounded in shape—which were thus developed in serum treated in the manner mentioned above, were in truth the rudimentary forms of a vegetable substance, in many respects very similar to that found in certain fluids after the act of fermentation. Having ascertained the existence of this vegetable in the serum of the blood, they extended their examination to other albuminous fluids, as the white of the egg, various morbid effusions, the serous part of purulent matter, &c.; and in all these—their alkalinity being first neutralised by the addition of an acid—the microscopic vegetable was discovered. The description which they give of their experiments is as follows:

"Fresh and pure serum of blood, after being rendered slightly acid by very diluted sulphuric acid, was mixed with about twice its quantity of distilled water. The liquid, at first quite transparent, becomes almost immediately cloudy in consequence of albuminous-looking matter becoming suspended in it. This amorphous matter gradually falls down to the bottom of the vessel, and collects there as a greyish-coloured sediment, while the supernatant liquor recovers its transparency, which it continues to retain, although very curious phenomena are soon developed within it. If, at the end of about twelve hours or so, a drop of this fluid be placed on the object-glass of a microscope, we observe diffused through it a number of vesicles, of a spherical or oval shape, quite independent one of another, and all perfectly diaphanous. Some of them appear to be empty; others seem to contain a sort of amorphous 'semis;' while a third set are observed to inclose a few distinct globules irregularly dispersed within their transparent cavities. It is always at the surface of the fluid, where this is exposed to the action of the air, that these vesicles are found to be first developed. Other changes are not long of making their appearance. The surface of these bodies soon begins to push out buds, which are similar in appearance and contents to the parent vesicles themselves, and which gradually lengthen into stems—these again giving out branches, and the branches giving out smaller ones, to an almost indefinite increase. All these divisions and subdivisions terminate in culs-de-sac or blind ends, so that the entire individual forms but one cavity closed at every point.

"Hitherto we have described the vegetable as formed, at its point of development, of a single vesicle, from which are given out buds, stems, and so forth; but there is another mode of formation which we shall now endeavour to explain.

"Instead of remaining solitary, the vesicles may be agglomerated in groups of twos or threes together, so as to constitute a complete system. From the simultaneous development of all these vesicles are produced hollow stems; some of which, as they increase, assume a moniliform appearance; while others retain a cylindrical shape, the hollow of the cylinders being divided into compartments by transverse septa. These new individuals, generated by the fusion of several vesicles into one, always terminate, as in the former instance, in blind culs-de-sac, and are observed to be either empty or partially filled with globules or with amorphous 'semis.'"

The phenomena now described are observable in the serum for four days or so, after it has been treated with the sulphuric acid. During this time, the fluid has become more or less completely turbid from flocculi of mucilaginous matter becoming suspended through it. These flocculi, if examined with the microscope, exhibit an inextricable meshwork, formed by the crossing and recrossing in all directions of the stems and branches of the vesicles, in the midst of which (meshwork) are observed numerous vesicles in different stages of development. At about the end of the fourth day, a new series of phenomena is observable; and what our authors have called the second period of development may then be said to commence.

"The surface of the fluid becomes covered with irregular patches, like floating islands, which the naked eye might take for accidental impurities deposited by the atmosphere. By the microscope, however, we find that they are composed of innumerable vesicles of various sizes, and very variously arranged—in some places, irregularly and at random; in others, with greater or less symmetry in straight or curved lines, or in the manner of arborisations.

"Amid this serum, which is found to be composed of an accumulation of genuine germs, and in the most superficial layers of the fluid, we are not long of detecting all those vegetable forms which were observed during the first four days, but which are now less simple in appearance and more varied in character. About the twelfth day, the entire surface of the fluid is covered with a thick membranous crust, which adheres to the sides of the vessel, and is formed by the interlacement of the numerous vegetable productions that have become developed.

"Beneath this serum, the fluid contains a multitude of vesicles and vegetable forms in different stages of evolution. If the serum be removed, another layer is speedily formed; and this phenomenon is repeated several times, until the whole fluid becomes putrid. We have seen this process of production continued for at least a month; at a certain period, spots of mouldiness appear on the surface of the membrane."

MM. *Andral* and *Gavarret* proceed to shew that the same phenomena are observable in other albuminous fluids, and conclude their observations by stating: "that, whatever be the origin of the albuminous fluid, and whether it be the product of healthy or of diseased action, if we only render it slightly acid and then dilute it with distilled water, we shall find, with the aid of a microscope, that an infusory vegetable becomes developed in it, under the influence of the oxygen of the atmosphere."—*Gazette Medicale.*

---

### MEMORANDA ON PHLEGMASIA DOLENS.

Although there is nothing novel or very striking in the following observations of M. *Tessier*, one of the physicians of the Hôtel Dieu in Paris, there is a practical tone about them which renders them acceptable. The author, like most of his countrymen, is apt to adopt exclusive opinions upon any subject to which he directs his especial attention—the favourite one of M. *Tessier* being the disease known by the names of purulent cachexy, purulent infection, purulent resorption, &c. and which, after all, seems to be close akin to, if not identical with, hectic fever.

*Case* 1.—A woman, who left the Maternité Hospital on the ninth day after her confinement, caught cold, and was seized with a sharp pain in the left side, which induced her to enter the Hôtel Dieu. By the use of cupping, &c. the symptoms were soon relieved; but then a painful swelling of the upper part of the right thigh supervened: the tumefaction gradually extended over the entire limb, from the hip down to the very foot. There was considerable pyrexia present, but no symptoms of any gravity. Under the use of very simple means, the swelling and pain left the right thigh, and settled in the left one, following exactly the same course as it had done before. At first there was great tenderness in the groin, extending along the inner side of the thigh, in which part a hard somewhat knotty cord could be distinctly felt: this was unquestionably the saphæna vein. The woman ultimately recovered perfectly. Subsequently the crural nerve became the seat of neuralgic suffering, and occasionally there was a tendency in the pain to settle in the abdominal parietes. Repeated purgatives were given with the view of preventing a localisation of the disease. (We should have preferred the use of warm baths, perhaps sarsaparilla, quinine, &c.—*Rev.*)

*Remarks.*—This was a mild case of the disease; as indeed most of those are which come on two or three weeks after delivery. The most dangerous cases usually supervene at a much earlier period, and are generally ushered in with shivering and other unpleasant symptoms. The puffy swelling of the limb too is almost always much greater; it is usually accompanied with more or less erythematous redness of the parts, and has a strong tendency to terminate in suppuration. The two sets of cases therefore are very different, and require very different modes, or perhaps we should rather say degrees, of treatment. In the one, simple emollients and febrifuges will usually suffice; but in the other, much more vigorous and energetic means must be used,

Of late years it has been the fashion with many writers to substitute the term *phlebitis* for the old one of *phlegmasia dolens.* This is certainly not right; as it is not invariably or necessarily the vein or veins that are chiefly affected. The disease in some cases is seated in the lymphatic vessels; while, in others, it seems to be confined in a great measure to the cellular tissue. When the case is really one of phlebitis, we can generally feel a hardened cord or cords along the inner side of the limb, and the disease is observed usually to commence with an œdematous swelling of its upper extremity, just below the point where the vein has become obstructed or obliterated. This arises from the circumstance that the blood, in the veins lower down in the limb, finds a way through anastomosing branches, whereas at this point it meets with an impediment.

In phlebitis, as in arteritis, the first phenomenon of the inflammatory process—of whatever character this process may be, suppurative or resolutive—is always the formation of a coagulum within the vessel. If suppuration occurs, the question at once arises, where is the pus formed, on the surface or in the substance of the clot? In ordinary phlegmon we know that this formation always goes on from the centre towards the circumference; and we may therefore presume that, in cases of suppurative phlebitis, the matter will be formed in that part of the vein corresponding to the middle of the coagulum. Authors have differed not a little among themselves on the point under consideration. Some have contended that the pus is secreted by the inner surface of the vein, and becomes effused within its tube; but then the difficulty arises, how can any effusion take place, seeing that the tube is completely obstructed by a coagulum? Others have said that it is secreted by the membranous envelope of the coagulum; but then we ask where is this envelope? We cannot see it. If we examine carefully for ourselves the phenomena in question, we find that the pus is situated in the centre and on the periphery of the coagulum; sometimes, but not commonly, between the two. We cannot suppose that imbibition has anything to do with this, as there is no matter to be found in the intermediate points. The pus is therefore contained in the cyst, so to speak, of the coagulum, and is thus prevented from commingling with the current of the adjoining circulation. There is always, in this case, an adhesive phlebitis, which precedes and sets limits to the inflammatory process. When pus is found on dissection blended with the mass of the blood, as for example in cases of glanders, &c. it does not proceed from any one vein that has become inflamed, but is 'formé de tout pieces dans la circulation'—*i. e.* is a spontaneous and intrinsic product of the circulating fluid.

*Case* 2.—A woman was twice bled during an attack of acute rheumatism. After the second operation, the arm became swollen and painful. In the course of a few days, a small quantity of pus could be made to ooze out from the wound, by pressure along the course of the cephalic vein. The swelling extended upwards to the axilla; and alarming constitutional symptoms—repeated shiverings, yellowness of the surface, thirst and dryness of the tongue, delirium, diarrhœa, &c.—supervened. The diagnosis was obvious; we had to deal with a case of traumatic phlebitis occurring during the course of a rheumatic attack. A number of leeches were applied on the arm night and morning, and it was kept continually in a bath; at the same time, quinine was administered in frequently repeated doses. Under this treatment the constitutional disease subsided in the course of a few days, and the local symptoms, with the exception of the swelling, were much abated—in short, a simple œdema was now substituted for a diffused phlegmon. After the lapse of ten days, all the symptoms had entirely ceased; but then a painful swelling of the mamma of the same side came on; fluctuation became perceptible in it, and the abscess, although rather deep-seated, was accordingly opened. Eventually the patient left the hospital quite well.

M. *Tessier* in commenting upon this case, considered it "an example of phlebitis, symptomatic of a purulent condition of the system," and contended that it is impossible to give any satisfactory explanation of the phenomena in such like cases upon any mechanical theory of a mere admixture of pus, secreted from an inflamed vein, with the current of the circulation. How, for example, can we thus account for the sudden appearances of those phlegmons and abscesses which are often so rapidly developed, and go on either to suppuration or to resolution in the space of 24 hours or even less, and all too without any previous inflammatory process?

In a practical point of view, the consideration of these phenomena is not less important. If, under such circumstances we attempt to arrest the progress of suppuration by making compression along the *trajet* of the inflamed vein, the chances are that an extensive diffused suppuration—similar to what we so frequently observe in the surgical wards—will be the result. It is often quite impossible to give a satisfactory explanation of the manner in which the particular symptoms are engendered; and therefore the only rational mode of treatment consists in accommodating our remedies to the indications presented by each case.

*Case* 3.—The secretion of milk suddenly ceased in a woman soon after her confinement, and symptoms of meningitis quickly followed. These soon gave way under active treatment; but then the patient was attacked with general feverishness, accompanied with cough, the expectoration of rusty sputa, and other signs of pneumonic inflammation. This was succeeded by a slight attack of peritonitis; which was followed in its turn by the occurrence of a *phlegmasia dolens.* Now this succession of morbid actions, or rather of different localisations of the same disease, was observed to decrease progressively in intensity from the meningitis in the first place, to the inflammatory affection of the lower extremity, which eventually terminated by a slow resolution.

It is not unfrequent to observe the very reverse of what happened in the present case; viz. where the metastasis of the disease takes place from the less to the more important organ, and ultimately proves fatal by the invasion of some vital part. A knowledge of such various localisations of the same morbid affection is of great importance for the right history, as well as for the successful treatment, of many cases of disease. Where a medical man has reason to suspect such a diathesis or state of system, his great object will necessarily be to keep the disease away, if possible, from those organs that are essential to life. As a remark of general truth, it may be stated that bleeding should be used under such circumstances with much greater reserve than usual, and that blisters and other external irritants are of more than ordinary utility.

In conclusion, M. *Tessier* frankly acknowledges that he cannot suggest any satisfactory explanation of these metastatic localisations of the same disease. Of this, however, he feels confident that the phenomena in such cases are certainly not owing to the absorption and mechanical admixture of purulent or other morbid matter with the blood, as is alleged by a number of writers in the present day.—*Gazette des Hôpitaux.*

*Remarks.*—It is very probable that M. *Tessier* carries his peculiar views on the subjects of phlebitis and purulent infection of the system too far; but it appears to us abundantly obvious that he has quite succeeded in showing that all the symptoms of the latter formidable affection may occur without the necessary co-existence of inflammation in any vein or veins. The cause of the disease in such cases is probably to be sought for in some morbid change of the entire mass of blood from the introduction of some deleterious matter, whether in the form of aerial miasm, or of poisonous fluid. In whatever disease there is a tendency to the occurrence of metastasis, or translation of the morbid action from one organ to another, we may reasonably, it seems to us, suspect the existence of some alteration or another in the blood itself. Is it not so in rheumatism?—in gout?—and again in that very class of diseases of which we have been treating in the present article?—*Rev.*

---

### Important Letter on the Contagion of the Plague.

This communication, coming from so respectable a quarter as that of the Director-General of the Quarantine Establishments of the Turkish empire (M. *Robert*) and one of the physicians of the sanitary council (M. *Marchand*), will be read with more than usual interest.

"In the Medical Gazette of last October (15th), there is an account of a work on the Plague, which its author, M. *Salle*, had submitted to the Royal Academy, and wherein he endeavoured to prove the non-contagiousness of the disease, and the consequent inutility of lazarettoes. For this purpose he has brought forward many assertions which truth compels us to say are utterly unfounded.

"Employed in the sanitary establishment of the Ottoman Empire, it appears to us a duty, which we owe to the profession, to rectify the statements which M. *Salle* has so authoritatively made, and to shew that the data on which he has based his opinions are not at all trustworthy. For example, he says; 'That the last two years have been in all respects similar to the four or five preceding ones; that the plague rages regularly in Lower Egypt and Syria, in spite of the most strict quarantine regulations at Beyrout and Alexandria; and that, on the contrary, the rest of the Turkish dominions, and especially Constantinople and Smyrna, have been quite exempt from the pestilence, notwithstanding the notorious negligence with which the quarantine establishment has been conducted.'

"To this assertion we answer that, if the plague has raged for several years past in lower Egypt and in Syria, the cause will be found in the utter absence of a well-organised sanitary code in these two provinces, more especially in Syria, where indeed there was no regular quarantine service before that country again came under the legitimate sway of the Sultan. That it continues to prevail there, is most probably owing to the still unsettled state of the province, and to the consequent difficulty of establishing, all at once, a system of prohibitory regulations affecting the transit of goods and the intercourse of the inhabitants.

"On the contrary, the other parts of the Turkish empire, and chiefly Constantinople and Smyrna, owe their exemption from the plague to the strictness with which the quarantine laws are carried into effect. During the course of last year, the pestilence was brought into the lazarettoes of these cities by ships from Alexandria, but was fortunately extinguished by the use of proper precautionary measures.

"The same remark may be made of the lazarettoes in Candia, Rhodes, Cyprus, the Dardanelles, and Salonica; in all of which places, the health has been more or less directly compromised by the arrival of infected vessels from Egypt—but, in each case, without wider diffusion of the pestilence.

"M. *Salle*, in a subsequent part of his work, says that, 'all the world knows that the young Sultan *Abdul-Medgid* has always set the *quarantinaires* at liberty, and treated them as he would do common prisoners, to whom he shews his royal favour on the occasion of any happy event.'

"This statement is utterly devoid of truth, as every official person at Constantinople can amply testify. It is true that, in 1839, when the sanitary service was imperfectly organised, and during the last moments of the late Sultan *Mahmoud* of glorious memory, a few days grace was granted to those who were then detained in the lazarretto of the capital; but, since that time, there has been no one instance of the remission of the established regulations in favour of even the most distinguished persons, as Muhib-Effendi and Rifaut Bey, or Said-Pacha, the son of Mehemet Ali, or Moustapha Pacha, the present minister of war. These and many other persons of not less note have remained in the lazarretto the full time appointed by the rules of the quarantine service.

"As to the notorious negligence with which this service is conducted, according to M. *Salle*, it is now, perhaps, scarcely necessary to say much. If, instead of drawing his information from uninformed or prejudiced quarters, he had looked into things with his own eyes, and, if, at the same time, he had been less presumptuous towards his correspondents in the East, he would doubtless have arrived at very different conclusions. As many physicians, however, who have resided amidst the plague, are and have been anti-contagionists, we are not, as a matter of course, at all surprised that M. *Salle* should hold the same opinion.

"But we have no hesitation in saying that it is our strong and decided conviction—a conviction that is founded on no little experience of the disease—that the immunity in the present day of many places in the Turkish empire (which a few years ago were not unfrequently visited with the pestilence) is entirely attributable to the system of quarantine regulations that have been recently adopted.

"In 1833, when the sanitary service was first definitively organised, the plague was then existing at Rousteink, Totrakan, Silistria, Philippopoli, and at Choumla and Varna—towns in European Turkey, where, although the same local and atmospheric conditions continue to exist now as they did then, the disease has not been known—thanks to the precautions that have been taken—during the last three years. The result of this pleasing change has been that the Austrian government have greatly relaxed the rigour of their quarantine on this frontier, and now grant free *pratique* to travellers, &c. coming from any part of Turkey in Europe.

"We may also enquire, how comes it that in our own country Marseilles, which has often suffered so severely from the pestilence, has been quite exempt from it, notwithstanding the constant communication which it has with different parts in the East, ever since it had a well-established quarantine? and that Greece, too, should have so well escaped of late years, since it has become an independent kingdom?

"Let it not, however, be supposed, from what we have now said, that we approve, in all respects, of the existing quarantine regulations of different European countries. While we contend that it is by them alone that we can reasonably hope to arrest the devastations of the plague, we willingly admit that a reform of the sanitary administrations is generally wanted, and that much of the rigour of the existing rules might be abated with no risk to health, and with great benefit to general commerce."—*Gazette Medicale.*

---

### On the Health of the Lazaretto at Alexandria, during 1842.

M. *Grassi*, head physician of this establishment, has published a report of its sanitary condition during the past year: from it we derive the following particulars:—

Although the number of persons attacked with the pestilence this year has been much less than during the preceding one, the severity of the disease and the ratio of the mortality have been nearly the same. The treatment, too, pursued was very similar as on former occasions, with the exception of bleeding being discontinued; both Dr. *Grassi* and Dr. *Colucci*, his colleague, finding it rather hurtful than otherwise.

Of 97 plague patients admitted into the hospital, 62 recovered, and 35 died: of the latter, 14 sunk after being seen once, and 1 died during the period of convalescence, from a complication of internal lesions. Of 166 patients attended at their own homes, no fewer than 151 died—a very great mortality; attributable no doubt to the unfavourable circumstances in which the sick were placed, and the want of the many advantages which are found in a hospital.

The course of the plague at Damietta, too, affords another striking confirmation of the circumstance now mentioned. None of the patients in this city were in such a position as to oblige them to go into the hospital; and hence, of 97 persons attacked and attended at their own homes, only 26 recovered.

M. *Grassi* appears to be strongly convinced that the plague is not so often spontaneously engendered in—whether from the overflowings of the Nile, the climate or soil of the country, the habits of the people, &c. as many writers have alleged—as it is conveyed to, Egypt, and then diffused by contagion from one sick person to another.

"It is mathematically demonstrated," says he, "that, about the middle of the year 1834, it was imported into this city (Alexandria)—at that time, and for nine years before, perfectly healthy—by a Greek vessel with passengers from Cyprus and Jerusalem, where the disease had been prevailing. Since this period, it has been reproduced here every successive year, simply because the germ of the poison has been retained in one or two spots, from which it has extended itself elsewhere."

M. *Grassi*, like M. *Robert*, (whose opinion we have given in the preceding article) is quite assured, in his own mind, of the great contagiousness of the plague, and that the only hope of keeping it out of any district is by a judicious system of quarantine regulations. "If," says he, "it follows from the statements now made that, even after all necessary precautionary measures of sequestration have been taken, the plague not unfrequently is observed to re-appear in a place, it deserves to be remarked that (according to the results of my observation) it has never broken out after the measures, which have been pointed out, have been enforced for five days. From this fact, we might infer that the period of incubation of the disease does not exceed this period of time—an inference that agrees with the opinion recently announced by M. *Auber* at the Academy of Sciences."

To avoid as much as possible the chances of the physician himself conveying the poison of the disease from the Lazaretto to the inhabitants of the city, the following precautionary measures are adopted:—"Before entering the hospital, we have to pass along a terrace that is assiduously watered. All the stairs too are kept in the same state. The wards, in which the patients are lying, are perfumed and continually ventilated by means of numerous windows in all the sides of the building. The beds are kept considerably removed from each other; and the physician, accompanied by the apothecary, visits each *at the legal distance;* an officer of the establishment being always in attendance to see that all the sanitary regulations are strictly attended to."—*Il Filocamo.—(A Maltese Journal.)*

---

### ANTHRAX CAUSED BY THE CARBUNCLE IN CATTLE.

The following remarks are from the pen of Dr. *Muller* of Hombourg, who, in his opening paragraph, says, "For two and twenty years I have been practising in a district where anthrax, arising from the carbuncle in cattle, is of such frequent occurrence that the number of cases which I have treated exceeds a hundred."

The disease in the human subject has almost invariably been observed in those seasons, when horned cattle were affected with the carbuncle, or what some authors have called the "malignant pustule." I have seen only one case in which it was attributable to the application of the nasal mucus of a diseased horse: this occurred in a groom, who nearly lost his life from the consequences that ensued. In the majority of my cases, I have been able to trace the operation of direct infection—usually in the hands of the peasants—from the contact of the blood of the diseased cattle, during the process of skinning them. In a few cases, the disease arose from a slight wound of the finger, while bleeding the animal during life; and in others from the pricks of gnats and other insects, which had previously been settling on the dead carcasses. On no occasion did it ever seem to be produced by merely eating the flesh of the diseased cattle.

The early symptoms are usually very simple. The person observes a small pustule over the affected spot; this is accompanied with a feeling of slight heat or itching in the part, but nothing more; and for several days there is probably no change. But then the surrounding cellular tissue begins to be affected with a diffused hardness, and the integuments become covered with numerous pustules, which are at first white and more or less transparent; then opaque, and of a yellow colour; and lastly put on a livid appearance. The induration increases, and the tubercle, always diffused, becomes larger, until it acquires the size of a pigeon's egg, or even sometimes of a man's fist. The character of the surrounding swelling is in part œdematous, and in part erysipelatous.

It has been usually at this stage that my patients have applied for advice, so that I have not often been able to use the caustic with the view of destroying the characteristic tubercle, while it was yet small—a plan of treatment which, on more than one occasion, has perfectly succeeded in my experience. I have never practised the excision of the part, as recommended and adopted with great advantage, we are told, by Dr. *Wetzer* of Bonn.

The most dangerous seat of the malady is the face; and unfortunately this is of by no means rare occurrence. I have seen many patients die from the effects of the swelling extending inwardly to the throat, and causing suffocation, before any typhoid fever had supervened. Often in those alarming cases, neither bleeding from the arm—which is frequently of the greatest service, if resorted to sufficiently early—nor deep incisions in the swollen parts, so as to cause profuse bleeding, will avail anything; and the medical man has the miserable task of seeing his patient asphyxiated, without being able to relieve him. (Might not tracheotomy be practised with advantage?)

Dr. *Müller* says that it is usually a favorable symptom when the centre of the tumour becomes gangrenous; for then the surrounding tumefaction—which, when the face is the seat of the disease, often extends up along the scalp, and down even to the chest—generally subsides more or less, and the pain is considerably relieved. If a line of demarcation is formed around the sphacelated portion of integuments, the case will, in all probability, do well. In general, the formation of sinuses (fusées de pus) beneath the dead cellular tissue has in my practice been prevented by the use of timely incisions, and by the removal of the gangrenous parts as early as possible. In one case, where the adoption of these means was delayed, it was necessary to make a counter-opening over the top of the sternum for a carbuncle on the cheek; and, in another case, at the elbow for one on the hand. In the latter case, the arm became almost as large as the thigh of the patient, and the swelling extended even to the integuments of the chest, so that the patient experienced much difficulty and distress in breathing. In both cases, however, the swelling quickly subsided after the gangrene had made its appearance; and ultimately a complete cure was affected. In the second one, the entire surface of the arm up to the shoulder was covered with livid spots, like large petechiæ, which yielded to the application of warm bread poultices prepared with a concentrated decoction of cinchona. Although the local disease was so formidable, the attendant fever was not very severe, and not of a typhoid type; while in other cases, in which the local affection was much less threatening, the patients have sunk under the constitutional disturbance.

*Treatment.*—Our author very distinctly says, that in scarcely any case did moist applications answer well. Poultices usually increased the swelling of the parts; while dry herbs in a bag appeared to agree much better. We have already said that, if the infected spot be excised or destroyed with caustic potash sufficiently early, the future mischief may very generally be prevented. When the gangrenous process has been fairly established, it is almost always proper that one or two deep incisions should be made—as in the treatment of ordinary spontaneous anthrax—in order to give issue to the purulent matter and sloughy cellular texture underneath, and thus obviate the formation of sinuses in the neighbourhood. "Since I have known," says Dr. *Müller*, "the utility of chlorine (chlore) against the effects of the bites of serpents, I have tried it in severe cases of carbuncle, and always with good consequences. In some of the worst cases that I have seen, I have trusted to the use of chlorine, after an emetic, (which he generally administers at the commencement of the treatment,) and have had ample cause to be well satisfied. The dose, which I usually give, is from half a drachm to a drachm every hour or two, either alone or mixed with water or other vehicle. As an external application, I employ an ounce of the chloruret of lime with four or six ounces of chamomile flowers, introduced into a bag, which need not be heated when applied: it usually retains its virtues for two or three days, as the odour will testify.

I do not propose these remedies as specifics; far from it. All that I say is, that they have proved more useful in my practice than any others which I have ever employed."—*Annales de la Chirurgie.*

---

### Interesting Case of deep-seated Abscess in the Groin.

In the last number of this Review, p. 550, the reader will find the history of some interesting cases of suppuration occurring in unusual situations, by Mr. *Henry James Johnson*. The following one is in some respects not unlike No. 4.

A middle-aged man, of a healthy constitution, and who had never exhibited any symptoms of coxalgia or spinal disease, was suddenly seized, while in the act of stooping down to lift up a burden, with a sharp pain in the right groin. The pain did not last above a minute; but it was followed by a sense of a dull and deep-seated uneasiness in the part, extending downwards for some way along the inner side of the thigh, and upwards in the direction of the lumbar vertebræ. Flexion of the thigh on the pelvis increased it; and the general movements of the limb were considerably impeded. The man's health now becoming not so good as it used to be, and the local uneasiness not mending, he entered the Hôtel Dieu under the care of M. *Roux*. At this time there was a firm swelling, of about the size of the fist, in the groin, apparently right under Poupart's ligament. The symptoms were obscure, and it was not easy to determine exactly the nature of the case. The patient however, after being some time in the hospital, was seized with a shivering fit; and next morning the swelling was found to be more diffused, and an indistinct sense of fluctuation was perceptible in it. The question now for consideration was, where was this abscess exactly situated? Was it in the sheath that envelopes the psoas muscle and the crural vessels and nerves? or was it connected with the sheath of the spermatic cord? and, if the former were the case, was there any reason to suspect the presence of lumbar disease? After well weighing all the circumstances of the case, it was deemed most probable that it was an idiopathic abscess situated in the sheath of the psoas muscle, and caused by the accidental rupture of some of its fibres, at the time that the patient experienced the sharp pain in the groin. After the lapse of a few days, the swelling acquired a larger size, and was then freely opened with the knife: it continued to discharge pretty freely for about a fortnight. At this time the integuments over the trochanter began to become swollen and puffy, and it was therefore suspected that the abscess might extend in this direction. This was found to be the case by introducing a probe into the wound in the groin; the instrument passed down deep among the muscles, so that its point could not be felt outwardly. It was deemed prudent that a counter-opening should be made, to prevent the burrowing of the matter. The incision was necessarily deep, to reach the extremity of the sinus: a large quantity of matter flowed out, when the opening was made into it. A tent was introduced to prevent the adhesion of the edges and keep the passage free. In the course of a few days, the discharge ceased from the wound in the groin, and came only from the one over the trochanter. It gradually decreased in quantity, and everything promised a speedy cure at the time when the report of the case was published.—*Gazette des Hôpitaux.*

---

### M. ORFILA ON A NEW ANTIDOTE OF CORROSIVE SUBLIMATE.

M. *Mialhe* recently announced to the Royal Academy of Medicine that, having taken a solution of the corrosive sublimate into his mouth, he found that the disagreeable taste was almost immediately dissipated by washing his mouth out with some proto-sulphuret of iron recently prepared and mixed with water. From this circumstance he inferred that the latter substance might act as an antidote of the former, and decompose it, giving rise to the chloruret of iron and the sulphuret of mercury—compounds which do not act poisonously on the living body. To ascertain the correctness of this opinion, I instituted, says M. *Orfila*, the following experiments.

*a*. I prepared 100 grammes of the proto-sulphuret of iron by decomposing the proto-sulphate of the metal with the hydrated sulphuret (sulfhydrate) of ammonia. The mixture was put into a large stoppered flask, kept constantly full of water, in order to avoid the contact of the atmospheric air—which would not have failed to have converted the proto-sulphuret into a per-sulphuret. The precipitate having all fallen down, I decanted off the liquid with a syphon, and then I filled the flask with water and corked it securely. When by repeated washings—always carefully excluding the contact of air—the liquid was found to contain no traces either of the sulphate of iron or of the sulfhydrate of ammonia, I gave a middle-sized dog about the tenth part of the proto-sulphuret suspended in water, and then immediately afterwards 60 centigrammes of corrosive sublimate dissolved in 100 grammes of water: the œsophagus of the animal was tied, and kept so for 12 hours. With the exception of several dejections from the bowels, it did not exhibit any of the usual symptoms of poisoning with the sublimate, and appeared on the whole to be very little inconvenienced, (not even with a ligature around its œsophagus?) On the next and following days, "il se portait à mearveille." A similar experiment was performed on another dog, and with similar results.

*b.* I introduced, by means of an elastic-gum tube, into the stomach of a dog, 60 centigrammes of corrosive sublimate, dissolved in 100 grammes of water, and immediately afterwards a quantity of the proto-sulphuret of iron similar to what was used in the first experiment: the œsophagus was tied, and kept so for 12 hours. The animal did not suffer more than in the first instance, and next day it had perfectly recovered.

*c.* The same quantity of the poison was given to another dog, and ten minutes were allowed to elapse before the dose of the proto-sulphuret was administered; in other respects the animal was treated as before. After the lapse of four hours, the ligature was removed from the œsophagus. The animal died in the course of the night, having exhibited all the usual symptoms of poisoning with corrosive sublimate. The conclusions I draw from these experiments are—

1. That the proto-sulphuret of iron completely neutralises the poisonous properties of corrosive sublimate, if it is administered in a sufficiently large dose immediately after the ingestion of the latter.

2. That, like the best accredited antidotes, it fails, if it be not administered for 10 or 15 minutes after the poison has been swallowed.

3. That—while we admit that the proto-sulphuret decomposes the corrosive sublimate much more energetically and efficiently than albumen does, and that therefore it should be preferred whenever it is at hand, so that it can be administered immediately after the poison—the medical man should never lose any time in waiting for it, but at once give the white of eggs blended with water freely in all cases of poisoning with this salt of mercury.—*Journal de Chimie.*

---

### Remarks on the Art of Diving.

It is well known to every one that, of late years, the art of diving and remaining under water for considerable periods of time has been attracting much notice in various countries of Europe. As the medical man cannot fail to take an interest in everything that appertains to human life, in the different situations in which men may be placed, a few remarks on this subject may not be unacceptable.

It is found that there is a great difference among men of equally robust and healthy constitutions as to their ability of becoming expert divers. Physical energy is not sufficient for this purpose; a certain moral strength and resolute equanimity appear to be of much greater importance. The six divers, who have been engaged in the wreck of the Royal George at Spithead during the last three Summers, were selected from the corps of sappers and miners as seemingly the best fitted for the purpose by their qualities of mind and body; but the effects of the submarine labour have been far from being alike in all. Some experienced a most distressing pain in the ears and a tendency to bleeding from the nose. Lieutenant *Hutchinson*, who superintended these operations, suffered so much in this respect that, although he made repeated efforts to overcome them, he never was able to remain for any considerable time under the surface. But, apart from the inconveniences felt at the time by submarine occupations, it has been very generally found by the men that their constitutional health and vigour have been very sensibly impaired, after they have been working for some time as divers. They become much weaker than they used to be, and are not fit for nearly the same amount of labour and fatigue, as formerly.

The season of their occupations is from May to October; and they are usually engaged eight or ten hours a day, remaining under water from half an hour to between two and three hours at a time. Most of their work is done between the full ebb and flow of the tide; at high water, the force of the stream is so great that considerable interruption is experienced. The flow of the tide is felt both more early and more powerfully at the bottom of the sea than at the surface; its force is often so great that it would upset the men, if they did not hold fast by some object near. When they come up, after having been down for an hour or so, they generally look pale and exhausted, although they may not feel so. They always wear flannel dresses under their water-proof clothes, to protect them against the cold to which they are necessarily exposed. As the air which they breathe through the tube, communicating between the helmet around their heads and the diving bell, is supplied to the latter by means of a powerful forcing pump, it is obvious that it, (the air,) in order to preserve a due equilibrium, must be at a pressure or condensation equal to that exercised by the superincumbent mass of water on the workman below. The quantity of the air thus transmitted by the tube greatly exceeds, as a matter of course, that which is necessary for respiration, and the superfluity escapes readily through a valve in the helmet into the sea. During the whole time that the diver is under water, there is a continual rising up of air-bubbles to the surface; this expenditure is as regularly supplied by the action of the forcing pump.

To enable a diver to descend with ease and rapidity to the bottom of the sea, he finds it necessary to carry a considerable weight with him. Besides wearing very heavy shoes, he has usually a thick plate of lead on each shoulder; and notwithstanding this extra-weight—amounting often to 120 or 130 pounds—he finds himself quite as light and unincumbered as only with his ordinary dress in the air. This is the natural effect of being placed in a denser medium.

The effects of the highly compressed air on the respiration have not hitherto been very satisfactorily examined by any physiologist; but it would seem that divers do not experience any decided difficulty of breathing, even at considerable depths. What seems curious, they find that they can sing (in a manner), but that they cannot whistle. They are able partially to converse with each other, by raising their voices as high as possible; the sound produced is then heard as if some one spoke in a very low tone.

As may be imagined, accidents are apt to occur from an interruption of the supply of fresh air or of the means of communication between the divers and the surface. Some time ago it happened that, when a man had been down for nearly an hour, the flexible air-tube, yielding to the great pressure on its sides, burst a little above the surface of the water with a loud whistling noise. One of the attendants immediately stopped the hole with his hand, and an attempt was made to draw the diver up without delay; but a minute and a half were lost before this could be effected. As soon as the helmet was taken off his head, it was found that blood was flowing from his ears, nose, and mouth, and that his face and neck were much swollen and discoloured; he lay in a semi-conscious or dozing state. There were large ecchymosed patches on the shoulders and upper part of the chest; and even the conjunctivæ of the eyes were partially infiltrated with dark-coloured blood. The patient vomited a little blood; his pulse was regular, and his breathing every now and then interrupted with deep heavy sighing. He was bled, and a turpentine enema was administered; the congestive symptoms gradually abated, and, in the course of two or three weeks, the man was nearly quite well.

Similar accidents have occurred on several other occasions. The phenomena exhibited in such cases evidently depend upon the circumstance, that if from any cause the pressure of the supplied air within the chest is suddenly withdrawn, that of the circumambient water on the parts of the body not protected with the helmet is no longer equipoised, and the blood is forced towards the head and neck with a power proportioned to the pressure, or, in other words, to the depth of the water. It was calculated that, in the case of the diver just alluded to, the pressure of the water on his body was equivalent to that of three atmospheres—a pressure which, before the occurrence of the accident, was counterbalanced by the condensed air, that was sent into the helmet by means of the forcing pump.

The recent experiments of Dr. *Payern*, which go to shew the possibility of remaining under water for many hours without the renewal of the air, have excited no little interest among scientific men. It still remains however to be determined how far they are to be depended on. From a notice of them in a number of the Revue Britannique, it appears that the means which the Doctor uses are very simple; viz. certain quantities of potash to absorb the carbonic acid evolved during expiration, and of chlorate of potash which at a moderate temperature gives out a considerable portion of oxygen.—*Gazette Medicale.*

---

### A simple Means to prevent or stop Nervous Coughing.

There is a curious and rather interesting paper with the above title in a number of the French Medical Gazette for February, and from it we have extracted the following passages. M. *Diday*, the author, makes a few prefatory remarks on the power which a mere strong effort of the will often gives to resist the impulse of a strong sensation, (*besoin*, the French would say,) like that of sneezing or coughing; and he goes so far as to assert that "it is always possible to prevent a fit of either by an energetic and sustained effort of the will, *if this be done from the very commencement*." In old established coughs, as a matter of course, such an attempt is quite inadmissible, at least with any reasonable hopes of success. Whenever, indeed, the cough depends upon the presenee of any matter to be expectorated, it is obvious that the effect cannot be checked as long as the cause remains. It is therefore almost exclusively to coughs depending upon nervous irritation that the following remarks are intended to apply.

The attempt also must be made before the annoyance has got, as it were, root in, or hold of, the system. At first, it is especially necessary that the patient acquire the habit of drawing in his breath slowly and not too deeply, and that he should avoid all hurried and irregular inspirations.

That most people have it in their power to arrest, at least to a certain extent, the frequent recurrence of fits of coughing and sneezing is obvious from the very circumstance that these acts are not unfrequently the effect of mere—we might almost say *wilful*—imitation; as in churches, lecture-rooms, &c.; and we know that, whatever may be acquired by the will, may be as readily checked by it. Dr. *Diday* tells us that one of the most eminent physicians of La Pitié hospital is in the habit of bawling out, on entering any of the wards, "I will have no coughing during my visit; whoever cannot stop himself, shall be put on low diet;" and true it is that all the patients are wonderfully still as long as his visit lasts. It is an old saying, that very few people know what they can do, until they try; and every one can testify from his own experience, that when the attention is suddenly engaged elsewhere, he ceases to feel a desire to some natural act, as to sneeze, to pass urine, &c., although this desire or *besoin* had been urgent just before.

M. *Diday* gives the following account of his observations.

" The first idea that occurred to me on this subject was suggested by what I observed to take place during sneezing. It is well known to many people that this act may be very often prevented by rubbing the edge of the eyelids or of the lips, or the tip of the nose, with your finger, when the disposition to sneeze is felt. I have repeatedly advised this expedient to my friends and patients, and it has almost invariably succeeded. In one case in particular, that of a young priest, who had been for some time much annoyed by the almost invariable recurrence of fits of sneezing that were apt to come on during his performance of mass, this simple means afforded a speedy and complete relief. In the performance of certain delicate surgical operations about the face and throat, such as that for hare-lip, fissure of the palate, &c., it is a matter of the greatest consequence that the patient should avoid all movement of the parts in the neighbourhood, and nothing disarranges everything so much as a fit of coughing or sneezing.

Now as the latter of these acts—which by-the-bye is nothing else but a cough of the nasal passages and of the back of the throat—may be so easily and effectually controlled in the manner we have just mentioned, we are natually led to expect the same in the former; and so I have found it to be the case not only in myself, but in several other persons who have tried the experiment under my direction.

By merely rubbing pretty smartly with the point of a finger the edge of the lips or of the eyelids, or the tip of the nose, when the first intimation of the 'besoin' to cough is felt, the act may often be entirely prevented. When this feeling returns very frequently, it will be found useful to employ the revulsive friction first on one of the parts, and then on the others in quick succession."

M. *Diday* says that the object in the rubbing is not to supersede the exercise of an effort of the will, but only to aid and augment its efficacy. He accounts for its effects on the principles of the excito-motory doctrines of *Marshall Hall* and *Müller* respecting the nervous system.

The impression, made on the extremities of certain branches of the trifacial nerve, acts as a sort of derivative or counter-irritant to the morbid sensibility of the extremities of the glosso-pharyngeal and pneumo-gastric nerves. We often observe the effects of this revulsive action in a somewhat analogous case, that of nausea; for we know that the disposition of the stomach to take on an inverted action may often be checked by rinsing the throat with a mouthful of spirits. If the stomach be not too much overloaded, this simple means will not unfrequently prevent vomiting. Here then is an instance where, by making a peculiar lively impression on certain sensory nerves, a tendency to muscular contraction —or, to use the language of a modern school, an excito-motory action—in another part, more or less removed from it, is prevented and controlled; and yet every one knows that a different kind of impression, that of titillation, made on the same organs, is one of the surest methods of exciting this very sympathetic action. Much, therefore, evidently depends upon the kind or degree of the irritation produced. While gentle tickling promotes, firm rubbing will often serve to check a sympathetic movement in a distant part.—*Gazette Medicale.*

### On the Difference of the Respiratory Movements at different Ages, &c.

We are not aware that the phenomena, to which MM. *Beau* and *Maissiat* allude in the following remarks, have been sufficiently dwelt upon in any of the recent works on physiology; and yet they are of that practical importance as to merit the attentive consideration of all medical men. The gist of their observations is that the type of the respiratory movements, or, in other words, the manner in which these are performed, varies not a little in the two sexes, and also at the different periods of life. The following abstract of their elaborate memoir will enable our readers to judge of its contents.

They describe what they call three distinct types of the respiratory movements, viz. 1, the abdominal type; 2, the inferior costal type; and 3, the superior costal type. In the *first*, the cavity of the chest is increased during inspiration, chiefly by the descent of the diaphragm and the protrusion of the abdominal muscles, the ribs being nearly motionless;—in the *second*, by the elevation and expansion of the lower six ribs, the movement of the upper ones becoming less and less considerable as we ascend, till it ceases altogether in the second and first;—and in the *third*, chiefly by the elevation and expansion of the upper ribs, the movement becoming less and less marked as we descend, till it nearly ceases entirely in the lowermost. In the second variety, the lower half of the sternum is much more moved, *i. e.* elevated and depressed, than its upper half; while the reverse of this is the case in the third variety.

In the majority of cases, each individual has, what may be called, his peculiar type of respiratory movements; and this seems to be remarkably constant at all times. One person breathes chiefly by the abdomen, so to speak; another by the lower ribs; and a third by the upper ones. A good deal however depends on age, and not a little on sex also, as we shall now endeavour to explain.

In the early period of life, and often indeed until three or four years of age, the respiration usually exhibits the abdominal type: the ribs are scarcely moved at all; and when the breathing is very much oppressed, as in many cases of pneumonia, the cartilages of the lower ribs are observed to be drawn somewhat inwards during each act of inspiration.

After this period of life, the three types begin to be marked in a more or less distinct manner, according to the individual's sex and constitution. The superior costal type is observed particularly in girls; while the other two types are found, in nearly equal frequency, in boys. As a person advances in life, we find that the first-mentioned type becomes more and more predominant in females; and one of the latter types more and more so in males.

The superior costal type in the female sex is readily recognisable not only in our hospitals, but in any assemblies of women, and more especially in actresses on the stage. Who has not observed that the upper part of the thorax, including the sternum and clavicle, is often, during the representation of strong mental emotions, most strikingly elevated and depressed? Nothing at all similar is ever witnessed in male performers. It may be supposed that this peculiarity in women is in some degree induced by the wearing of stays and the consequent compression of the abdomen and the lower part of the chest. But, although this absurd and most pernicious custom* may unquestionably increase the degree or amount of the peculiarity in question, it certainly does not give rise to it; for we

* To do all manner of justice to the ladies, we must frankly admit that our authors are by no means so much opposed to the use of corsets as most medical men are. "We may here state *en passant*," they say, "that the use of the corset is not nearly so hurtful to females, as is generally imagined. Indeed it find it to exist in girls before they ever wore stays, and in country-women who never wear them at all.

One important end gained by the respiration being superior-costal in the female sex is abundantly obvious—the breathing is thus much less disturbed than it otherwise would be during the period of pregnancy. This point, although seldom or never alluded to in modern lectures or writings, has not escaped the attention of some of the older physiologists. *Boerhaave*, in his Prælectiones, thus makes mention of it: "Hinc in fæminis inspirantibus sternum rursum et oblique extrorsum vertitur, totusque thorax quasi assurgit; hinc etiam tumente abdomine, liberius respirant." *Haller*, the great commentator of *Boerhaave*, is still more exact and minute in his description of the peculiarities of the respiratory movements in the two sexes: "Considerate puerum anni unius et puellam ejusdem ætatis in eodem lecto unà dormientes, videbis in puellâ, quando inspirat, totam thoracis molem ascendere versus jugulum; in puero verò inspirante thoracem et claviculas vix moveri. In viro adulto pectus vix movetur equidquam, dum respirat; in fæminâ totum sursum trahitur, ut â diaphragmate recedat. Ergo vir abdomine maxime respirat, fæmina thorace. Nisi hanc fæmina diversitatem natura fecisset, gravidæ perpetuâ dyspnœâ laboravissant æque ac viri hydropici."

It would seem that most of the authors of the present day have quite overlooked these interesting peculiarities in the phenomena of the respiratory movements, so well described almost a century ago. It has been generally supposed that the movements are nearly alike in all persons; and hence one of the chief objects of enquiry among modern physiologists has been to determine the *where* and in what direction the principal enlargement of the chest takes place. We cannot therefore be surprised at the difference of opinion on this point. While M. *Gerdy* maintains that the greatest enlargement of the thoracic cavity occurs in a transverse direction on the level of the lower ribs, we find MM. *Hourmann* and *Dechambre* infer from their observations—on the old female inmates of the Salpetriere hospital—that it is in the upper part of the chest, and that, "in this general movement of ascension, the sternum is, on an average, raised from eight to twelve lines." And what is the general conclusion to which they come? That "in proportion as a person advances in years, the motory powers of respiration—and more especially those which tend to increase the transverse dimensions of the chest—become considerably impaired in their energy." It is scarcely necessary to say that the mode of respiration, described by these gentlemen, is not the result of age, but is natural to the female sex. It is however much more conspicuous in young than in old women.

MM. *Beau* and *Maissiat* proceed to notice the peculiarities of the respiratory movements in different animals; for the type, so to speak, is far from being alike in all. In the rabbit, the horse, the cat, &c. it is chiefly abdominal; while in the dog, it is inferior costal in a very marked degree. In no animal is it superior costal, at least in the natural and healthy condition; and perhaps for this reason that the alternate elevation and depression of the upper ribs and of the sternum would have interfered not a little with the movements of the fore legs in running. This mode of respiration may therefore be regarded as peculiar to the human race, and more especially to the female sex.—*Encyclographie des Sciences Medicales.*

---

may almost be asserted that they are, so to speak, organised for the wearing of this piece of dress. (!) On the other hand, it is an absolute physiological absurdity (contresens) for men to wear stays; for their breathing is chiefly by the lower ribs and the abdomen."

### Pulmonary Emphysema: Discussion at the Royal Academy.

The pathology of this disease is still a subject of dispute among medical men; and therefore the recent discussion at the Royal Academy, in which many of the most experienced physicians of the French metropolis took a part, was attended with more than usual interest. The discrepancy of opinion has most probably arisen from the circumstance of certain writers alleging, or, at all events, seeming to imply that its proximate cause, or producing lesion, is always the same; whereas this unquestionably varies not a little in different cases. In some, it seems to depend on a mere abnormal dilatation of the pulmonary vesicles, (these having lost their natural elasticity,) or in a junction at the same time of two or more of these into one, in consequence of a partial rupture of their thin parietes. In others, along with this dilated state of the air-cellules, there seems to be a greater or less degree of thickening of their mucous lining—the result of chronic bronchitis, or, as it is usually called, catarrh; while in a third set of cases, the inspired air has become extravasated into the inter-vesicular cellular tissue of the lungs, in consequence, no doubt, of a sudden giving way of the air-cells from their over-distension. This last kind of pulmonary emphysema is almost always the result of a violent exertion, by which the respiration has been much embarrassed, and during which the inspired air has been forcibly held to enable the individual to put forth more than usual muscular strength. Hence it is more frequent in horses than in any other animal, or than in man himself; but in the latter it has been not unfrequently observed during some extraordinary effort of the body, or some violent emotion of the mind.

The two first-named kinds of the disease are of much slower and more gradual development. They are very frequently associated with some other lesion of the respiratory apparatus, or of the heart and great blood-vessels. Indeed whatever tends to embarrass the natural soft and regular play of the lungs is extremely apt to be followed by a greater or less degree of dilatation of the air-cells; and the reason of this is abundantly obvious.

The breath is often held much longer than it is in health;* the minute cells are more dilated than usual, in consequence partly of the air becoming expanded from the heat of the body; and thus the elasticity of the vesicles is gradually more and more impaired. If their mucous lining be somewhat thickened at the same time, and if there be also, as is generally the case, an increased secretion from its surface, we can readily understand the cause of many of the symptoms in old and obstinate lung complaints.

As will afterwards appear, objections were made to some of these positions by different members of the Academy, and we must admit that the objections in certain cases were very reasonable. However this may be, the conflict of opinions on any question of science can never fail to be attended with good, provided there be no unnecessary rambling of discourse, and no presumptuous indulgence in fanciful theories.

We have omitted to mention that the discussion arose on receiving a report, drawn up by M. *Adelon*, upon a paper of Dr. *Prus*, in which this physician endeavoured to shew that pulmonary emphysema is not unfrequently the direct cause of sudden death. On this point all the speakers—with the exception of two, the reporter and M. *Ollivier*—expressed their dissent from the opinion of Dr. *Prus*; the general impression being that this affection of the lungs, though

---

* M. *Collard* mentioned that he had seen emphysema of the lungs induced in young men, who were passionately fond of smoking, and had acquired the power of retaining the smoke of the tobacco for an unusual length of time.

often a most distressing and generally an incurable complaint, is seldom dangerous *per se*. The veterinary authorities took nearly the same view of the subject. M. *Barthelemy*, however, one of the highest, distinctly asserted that he had seen one, if not two cases, where sudden death in the horse was caused by an attack of pulmonary emphysema. The same may therefore be supposed to be possible in the human subject under particular circumstances; a rent takes place in some of the vesicles; the air is rapidly effused into the inter-lobular cellular tissue, causing great compression of the vesicles, and consequent serious embarrassment of the breathing. Now this is most apt to occur either during, or immediately after, some violent exertion, when—be it remembered—the heart is acting furiously and the respiration is hurried and laborious. Under such circumstances, we may readily suppose that a slight additional impediment will suffice to induce a rapid asphyxia.

It has been too much the fashion for the last twenty or thirty years, (when such exclusive attention has been paid to a purely *material* and visible pathology,) to attribute most cases of asthma to pulmonary emphysema, or to some other organic affection of the lungs. M. *Ferrus* exposed the fallacy of this idea with great ability, and pointed out that disordered innervation is one of the most important elements in the history of this disease. All unprejudiced pathologists must admit that it is often impossible to discover the slightest deviation from the normal structure of the lungs in those, who have long suffered from asthma. M. *Ferrus* is surely therefore quite right in regarding the disease as (often at least) a genuine Neurosis. He is also of opinion that, in the cases where sudden death has been attributed to emphysema of the lungs, a nervous affection of this sort had very seriously complicated this mechanical cause of disturbance.

"The symptoms" says a most judicious writer in the French Medical Gazette, "attending the morbid affections of the chest, as well as of other regions of the body, can be submitted, only within a certain limit, to the explanations deduced from mere anatomical or physical interpretations. Beyond this point, it is in the vital activity which animates every organ, that we must seek for the reason of most of the phenomena that occur in disease—unless we are ready to declare, with the mechanical physicians of old, that the living body is only an assemblage of hydraulic pipes, pulleys, and levers, or, with some modern wiseacres, to regard it as a mere perfect and admirably constituted steam-engine."

The most prominent symptoms of pulmonary emphysema are usually a hurried and embarrassed state of the breathing—in many cases, every now and then aggravated in paroxysms—and a dry troublesome cough; the auscultatory signs being a loud respiratory murmur, and the presence of sibilant and other dry *râles*. When air has become effused into the inter-vesicular texture, there is an unusual resonance of the chest on percussion.

From all this it is rendered highly probable that pulmonary emphysema is present, in a greater or less degree, in most cases of asthma or spasmodic dyspnœa—not indeed as the sole and essential element of the disease, but as one of several morbid affections that are very generally co-existent. We now give a very brief abstract of the more important remarks which some of the leading academicians made, upon the reading of the report on M. *Prus*' paper.

M. *Louis*.—I see no reason for believing that the disease, known by the name of pulmonary emphysema, is ever attended with danger to the life of a patient, however distressing and painful may be its effects. That sudden death may be produced by such a cause alone, I am scarcely prepared to admit: the co-existence of heart disease being of very frequent occurrence. Let it be well remembered that it is often exceedingly difficult to account satisfactorily for many sudden deaths, either by the symptoms during the life of the patient, or by the appearances discoverable on dissection. That the proximate or organic cause of pulmonary emphysema is an hypertrophied state of the pulmonary vesicles is certainly most probable: but the truth of this has not been positively demonstrated.

M. *Dupuy*.—It is now ten years ago since I proved by anatomical preparations that the seat of pulmonary emphysema is the inter-lobular cellular tissue of the lungs. By drying the lungs first, the dissection is rendered much more satisfactory. In many instances I have been able to shew most distinctly that there was an effusion of air 'hors des vesicules.'

M. *Bouillaud*.—I quite agree with M. *Louis* in his opinion that there is a close relation between emphysema of the lungs and organic diseases of the heart; the former is very frequently the effect or result of the existence of the latter. With respect to there being an hypertrophy of the air-vesicles in cases of pulmonary emphysema, we must admit that all analogy is strongly in favour of such an opinion. It is very often to chronic bronchitis that emphysema is consecutive; and, as it is the character of inflammation, wherever this be seated, to leave a thickened and hypertrophied state of the parts affected, we may reasonably suppose that such will be the case after an inflammation of the air-passages. My experience quite confirms another remark of M. *Louis*, that there is seldom any danger from pulmonary emphysema *per se*, and that, however alarming the symptoms may be for a time, seldom or never is any case of sudden death attributable to this cause.

M. *Rochoux*.—A great deal has been said of Hypertrophy of the pulmonary vesicles; but has any one ever seen this state of the lungs? we deem not; it is only by reasoning, by analogy, that its existence is presumed; *voila tout*. Emphysema by itself occasions nothing more than merely a certain difficulty and embarrassment in breathing. It will never do to attribute to *it* the many serious alterations, with which it is so frequently associated.

M. *Piorry*.—The most important question that we have to determine is this: when the pulmonary cells are much dilated, does the individual necessarily experience difficulty in breathing? But then we may ask, does not the air penetrate as well into a dilated cell as into one of the ordinary dimensions? and moreover, is it not the case that in ordinary breathing a part only of the lungs is called into play? Let us here observe that, in cases of emphysema, the *rale* is heard during the act of expiration, and not during that of inspiration; and moreover that the dyspnœa and other distress of the patient are always relieved by expectoration. In my opinion, the true cause of the embarrassment of breathing in emphysema is a constriction of the bronchial tubes; and certainly its most prominent symptoms are strictly in accordance with this view of its pathology. That sudden death should be caused by pulmonary emphysema alone seems to me to be scarcely probable.

M. *Renault*.—If the disease be of frequent occurrence in the human subject, it is still more so among horses. From a pretty extensive experience, I may confidently state that short-windedness is the most frequent and characteristic symptom of pulmonary emphysema during the life of the animal. I have never however been able to discover, even with the aid of the microscope, any appearance of thickening in the pulmonary vesicles. Emphysema not unfrequently occurs on a sudden and almost instantaneously: it is then the result of a rupture of the pulmonary cells, and of a consequent effusion into the interlobular cellular texture.* I have never known sudden death to be caused in such a way, nor

* There is manifestly a mighty difference between the dilated or—as it is most have I read of such a case. (M. *Barthelemy,* however, another veterinarian authority, subsequently stated that he had witnessed an instance of almost instantaneous death from this cause.)

M. *Ollivier.*—The opinion of most of the preceding speakers that sudden death cannot be produced by mere pulmonary emphysema is, I think, not quite correct. The following illustration of it occurred in my practice about six years ago. A youth received a blow from another in a fight; this so exasperated him that he rushed upon his opponent, and would have struck him down, had he not been restrained by the by-standers. He had scarcely walked forty steps from the place, when he suddenly fell down and expired. On examining the body next day, I was particularly struck with the circumstance, that when the cavity of the thorax was opened, the lungs projected fairly out beyond the ribs. This peculiarity was, it seems to me, owing to the effusion of air into the interstitial cellular tissue of the lungs. It is right that I should state that I knew nothing of the youth's previous health.

It appears to me that the greater number of sudden deaths are attributable to some lesion or another of the lungs. An interesting case, which occurred recently to me, may be worthy of notice. A man, in a fit of violent passion, fell down and expired. The only morbid appearance found on the dissection of the body was an excessively gorged state of the lungs, so that, on making an incision into them, the blood gushed out as from 'un boudin.'

M. *Blandin.*—Before we can accurately determine the anatomical seat of pulmonary Emphysema, we should require to know exactly the normal structure of the lungs; and yet there is no little discrepancy of opinion among anatomists on this subject. The question at issue among some of the leading authorities in the present day is, whether the pulmonary tissue be composed of isolated vesicles, or of cellules which communicate with each other. As well as I can understand the subject, it certainly appears to me most probable that there is a simple dilatation of the air-vesicles in cases of pulmonary emphysema. A circumstance, which seems to confirm this view of the question, is that we cannot in the dead body press the air out of the *ampullæ,* which constitute the emplysematous distension.

M. *Roux* enquired of M. *Ollivier* if, in the interesting case which he had related, there was no appearance of air having become mixed with the blood in any part of the body. He alluded to a curious case which he had seen in company with *Bichat,* nearly forty years ago. There was brought to the amphitheatre for dissection, a body, which exhibited all the marks of a sudden and perhaps violent death. On opening the cranium, the cerebral arteries were found to be distended in part with bubbles of air. It was afterwards ascertained that the man had died during a violent and prolonged effort. *Bichat* supposed that some of the pulmonary septa had given way, and thus allowed the air to escape and become blended with the blood.* M. *Ollivier* replied to the question that no such traces had been observed in his case.

---

absurdly called by most of the speakers—*hypertrophied* state of the natural vesicles of the lungs, and the effusion of air into their inter-vesicular cellular tissue, in consequence of an accidental rupture of any of the vesicles; and yet these two very different states seem to be confounded together, as if they were the same affection. The case, narrated by the next speaker, was evidently one of traumatic emphysema, and not of vesicular dilatation.

* It deserves to be noticed that there seems to be in some cases spontaneous evolution of gas, or at least a rapid dilatation of the air which exists in all dead

M. *Rochoux*.—By a singular confusion in medical language, the same term, Emphysema, is applied to two very different morbid affections: viz. the dilatation of the pulmonary vesicles on the one hand, and the infiltration of air in the cellular tissue between these vesicles on the other. It is to the first of these states that I would restrict the appellation. If it was kept in mind that pulmonary emphysema is almost always coexistent with other diseases, either of the lungs or of the heart, physicians would be less surprised, than they seem to be, that sudden death should occur under such circumstances. When air becomes extravasated into the inter-vesicular cellular tissue, it must have the effect of compressing not only the air-vesicles themselves, but also the free circulation in the blood-vessels, and thus tend considerably to embarrass the respiratory and circulatory functions. This element in the symptomatology has not attracted the notice that it seems to merit. If the compression be very great, we cannot be much surprised that death may sometimes ıapidly take place under such circumstances.—*Gazette Medicale*.

---

### Two or Three Scraps from Galen.

M. *Dubois d'Amiens*, a physician of high literary as well as professional accomplishments, and whose name our readers may perhaps remember in connexion with that of the late M. *Double* as an ardent admirer of the classic authors, has of late been contributing occasionally a paper to the L'Experience on that portion of *Galen*'s writings, which treats of the diseases of the nervous system. We have selected the following quotations and comments thereon, as a sample of M. *Dubois*' mode of treating his subject.

### *Prognosis in Apoplexy*

What says the old Roman on this important subject?

"We judge of the gravity of an apoplectic seizure by the degree of the disturbance in the respiration. Whenever this deviates much from the natural rhythm of its movements, it may be at once suspected that the lesion of the encephalon is very serious; if the deviation is inconsiderable, the lesion is most probably slight. But the worst of all states is, when the respiration is irregular and intermittent (entrecoupé) and is performed with violent efforts."

This reflection is drawn from the observations which the author had previously narrated. He had pointed out, from the difference in the seat of the paralysis, the localisation of the mischief in different parts of the cerebro-spinal axis; from the disturbance in the breathing, he had concluded that the lesion was seated above the cervical portion of the spinal marrow: and he had shewn that, if there was a paralysis of the face at the same time, it was infallibly in the encephalon itself.

Having elucidated these points, he next proceeds to discuss the subject of the prognosis of the disease, and shews with great ingenuity of reasoning, that the degree of the disturbance in the respiratory movements is by far the least fallible sign to be trusted to in this respect.

This opinion of *Galen* has been very generally adopted by subsequent writers on apoplexy; although lately M. *Rochoux*, in his work on the subject, has questioned its accuracy. But this gentleman seems to have mistaken the real expressions of the Roman physician, who nowhere attaches any over-importance

---

bodies, in consequence of speedy decomposition. This phenomenon appears to be more frequent in certain seasons and after certain maladies; especially when the death has been sudden, and the body is full of all the animal juices.

to the mere stertorousness of the breathing. That *Galen* is far from neglecting the consideration of other symptoms, independent of those exhibited by the respiration, will be seen by the following passage:—

"In the same manner as the existence of a paralysis of the whole body, with the exception of the face, indicates that the cause of the affection is seated at the origin of the spinal marrow, so the occurrence of general convulsions leads the physician to the same conclusion, provided always the face remains unaffected; but if *it* also becomes implicated, we many reasonably infer that the mischief has its seat higher up, viz. in the encephalon."

These two morbid symptoms, viz.—convulsions and palsy—are rightly associated together by *Galen*. He had distinctly perceived that they were both often attributable to the same cause; or, at all events, that they were generally connected with some lesion of the same part of the animal economy. To shew the truth of this position, we have only to select any part of the nervous centres, whether of the spinal marrow, or of the encephalon; then act upon it by irritating or lacerating it; and we find that at first convulsions, and afterwards paralysis, is the result of such an injury.

The remarks of the old Classic, on the mode of distinguishing a lesion situated near the top of the spinal marrow from one of the encephalon itself—the face being scarcely, if at all, affected in the first instance, and almost invariably in the second—are quite in accordance with the experience of the best physicians of modern times. No doubt, exceptions can be stated to their absolute correctness, and the results of post-mortem examinations do not always tally with the rules here laid down; but may not the same be alleged of almost every position or axiom in the history of disease? If such a remark holds true in a great majority of cases, this is all the degree of certainty that we can expect in medical matters.

The simplicity, too, of *Galen*'s language, in reference to the diseases we are at present alluding to, deserves some notice; he talks of the lesion, whether of the medulla or of the cerebrum, as an affection or suffering (*παθος*,) which has its seat in one place (*τοπον*,) or another of the nervous system. Contrast such language with that of *Willis*, and other authors of his date, and you find them descanting upon spasmodic particles affecting the brain, either from some *vice* of the blood, or of the organ itself; on the explosion of the *animal spirits;* on the alterations of the *nervous juice;* on morbid *humours*, &c.

Having described the leading features of distinction between encephalic and spinal diseases, *Galen* advances in his enquiries, and seeks to discover the exact part that is affected. His several positions are taken up with no ordinary philosophical caution as well as skill, and worked out with the greatest ingenuity. Take, for example, the following short paragraph.

"A part being convulsed, there is necessarily a morbid affection either of its motor nerve, or of its muscular substance, provided no part of the nervous centres be diseased."

Here, then, we have most clearly and emphatically enounced one of those important truths in reference to the pathology of the nervous system, which it is the boast of modern science to have discovered. The expression, *τὸ κινητικὸν νεῦρον* (the motor nerve,) alone suffices to indicate what clear ideas *Galen* had on the subject. May we not fairly infer, from the expression just quoted, that in his opinion certain nerves were exclusively and essentially subservient to motion, and others to the function of sensation? This, it should be remembered too, was founded on, and derived from, mere pathological observation; and we need not now say how completely its accuracy has been established by the researches of *Bell* and others in the present century.

After designating the two kinds of paralysis, by the terms *anæthesia* (loss of feeling) and *akinesia* (loss of motion), he candidly admits the difficulty of explaining the question as to their cause in the following paragraph:—'

"Both Herophilus and Eudemus, who have been the first since the time of Hippocrates to describe with accuracy the anatomy of the nerves, have left a great problem still unsolved, when they could not explain how it happens that in certain cases of palsy the feeling only, and in other cases the power of movement, is lost; while, in a third set of cases, there is an abolition of both faculties at the same time."

The important problem is here, it will be observed, most clearly and emphatically enounced; and one cannot fail to admire the candour with which the writer admits the difficulty of its solution, and his philosophic sagacity in avoiding any unsupported speculations on the subject.

In *Galen*'s time, as in our own, there were men who wasted their lives in disputes and dissertations upon *words*, without ever searching after the pith and marrow of *things*. He is unusually intolerant of these gentlemen, and repeatedly urges them not to lose their time in such pursuits. One insisted upon restricting the term paralysis to loss of motion; another to loss of sensation; and so forth. What does he say?

"I request you to allow each one to make use of such denominations as he thinks best; but let us, without ceasing, have for our chief object to find out the part that is the seat of the disease, and the nature of the disease at the same time. As long as we have not positive knowledge on these subjects, in vain can we expect to treat with success the lesions either of motion or of sensation."

No medical precept can be better, or better put, than this—to search for the exact seat of the mischief first, and then to try and make out its real nature.

The case of the sophist *Pausanias*, related by *Galen*, although it seems to have been a very simple one, shews how judicious his therapeutic ideas were.

"*Pausanias*, who had recently made a voyage from Syria to Rome, began to experience a loss of feeling in the two last fingers, and on one side of the middle finger, of his left hand: under injudicious treatment, the insensibility of the affected parts became complete. I made enquiries into his condition, and learned, among other things, that during the voyage he had fallen from his chair and struck with force the upper part of his back; the contusion was soon cured, but a numbness of the fingers supervened. I immediately advised that the same remedies, which had been applied to the fingers, should be applied to the part that had been first injured, and my patient speedily recovered the entire use of the left hand."

We observe here the practice of a skilful physician; not to regard alone the part that is more prominently and immediately affected, but to seek for the producing cause, and to find out whether the local lesion may not be dependent upon an injury elsewhere."—*L'Experience*..

---

### An Occasional Phenomenon in Phlebitis.

In a recent number of the Annales de la Chirurgie, there is a sensible paper on what the French writers call Purulent infection of the constitution, by M. *Sedillot*, Professor of Military Surgery at Strasbourg. He admits, with M. *Tessier*, that all the phenomena of this formidable disease may be occasionally present, and yet that no decided traces of inflammation in any vein may be discoverable on dissection: but such an occurrence is, in his opinion, the exceptional, not the general, case. Phlebitis, therefore, is certainly not the invariable and only cause of the cachectic state to which we are alluding. But while he thus objects to the doctrine of M. *Blandin* when pushed to its full extent, he expresses his decided dissent from M. *Tessier's* views, that purulent infection is *never* produced by the direct admixture of purulent matter with the blood, and that it is always attributable to a peculiar alteration of the vital fluid, which gives rise to the formation of abscesses and purulent effusions in different parts, as one of its effects and symptoms.

As we have repeatedly of late adverted to this disputed point in pathology, and in one of our recent numbers gave large extracts from two very able letters addressed to each other by the chiefs on either side of the question, we shall not pursue the subject farther at present, but merely select the following extract from M. *Sedillot's* paper on a peculiar phenomenon which has been observed in a few cases of phlebitic disease, and which is probably not well known to many of our readers.

"The venerable M. *Ribes* shewed many years ago (vide his Memoires et Observations d'Anatomie, de Physiologie et de Chirurgie) that an inflamed vein, after having become indurated, painful, nodose, partially filled with pus, and impermeable for some extent to the blood, may recover its healthy condition under a process of resolution, so as again to admit the circulation to pass through its calibre. This remark of M. *Ribes* has subsequently been confirmed by several pathologists, and its accuracy cannot now be disputed. How are we to explain the phenomenon? Doubtless in some cases we may believe that an interstitial absorption of the purulent matter has taken place; but in others may we not suppose that the enveloping or surrounding membrane of the abscess within the vein may burst or ulcerate into the adjacent portion of its canal, which has remained permeable to the blood? Why should that which is of daily occurrence in common abscesses—viz. their opening on the side where the resistance is the least—be impossible in the case of venous abscesses? In what other manner too, shall we explain the presence of purulent matter not only in the veins surrounding many abscesses, but extending from these even to the venæ cavæ and the heart itself, except in some such manner as I have hinted at?"—*Annales de la Chirurgie.*

---

### THE CONNECTION BETWEEN ENLARGEMENT OF THE SPLEEN AND INTERMITTENT FEVERS.

M. *Piorry,* the "plessimetric" physician *par excellence,* and who takes everything appertaining to percussion under his especial patronage, has been lately edifying the Royal Academy with his lucubrations on the intimate connexion, as cause and effect, between enlargement of the spleen and intermittent fevers.

According to his view of the question, the primary action of marsh miasms is on this abdominal viscus, and the direct result of this action is the development of paroxysmal fever. The reader may presume, as a matter of course, that the state of the spleen is to be ascertained by plessimetric percussion.* The following is a selection of the very numerous deductions, (upwards of 60 in number) drawn by the author from his experience during the last fifteen years.

1. According to the results of 165 cases observed by me, paroxysmal fevers of different types do not essentially differ in their nature, the one from the other. They have mutually succeeded the one to the other, although the appreciable organic lesion remained the same in all. The greater or less rapidity, with which

---

* We observe by the most recent numbers of some of the French Journals, that M. *Piorry,* having sufficiently dwelt on the pathology of the spleen, has been subsequently extending his plessimetric investigations to elucidating the diagnosis in various diseases of the kidneys. Probably each important organ of the body will have its turn.

the paroxysms return, does not materially influence the treatment required; the same means being found to succeed in fevers of dissimilar types.

2. In the present state of medical science, we cannot at all explain why a paroxysmal fever should assume one type rather than another—a tertian rather than a quartan, or a quotidian rather than a tertian.

3. Many authors have affirmed that quotidians are of much rarer occurrence than tertians or quartans. According to my experience, however, of 152 cases of intermittent fever observed in Paris, 98 were quotidians and only 54 of the other types.

(May not M. *Piorry* have on some occasions set down common ephemeral fever as genuine intermittent? Genuine quotidian ague is surely not of common occurrence in Paris.)

4. From the analysis of 163 cases, it is incontestible that other causes, besides the influence of marsh miasms, may give rise to intermittent fevers. Falls or blows on the left side, inflammation and organic lesions of the spleen, &c. have been known to be followed by such fevers; the type, under such circumstances, being usually quotidian. Mere percussion of the splenic region will sometimes induce the *cutis anserina,* and a feverish tremor.

(Here the hobby-horse begins to make his appearance.)

5. Intermittents of various types are generated in Paris from the operation of marsh miasms. The effluvia from the Seine, the Bievre, and the canal, and even from the turning up of the soil for the construction of drains and so forth, are abundantly sufficient for the development of the malady.

6. The frequency of hypertrophy of the spleen in these fevers is so great that it was found to be present in 154 out of 161 cases, in which the state of this viscus was attentively examined. In four of the remaining seven cases, it was painful on pressure; and as to the other three, M. *Piorry* observes that they were not seen by him, and it is probable that the finger, and not the plessimeter, was used in performing percussion.

7. In many cases, the splenic pain or uneasiness precedes the development of the fever, and seems indeed to be the 'point de depart' of it. It is highly probable that the pain is seated in the nervous plexus of the spleen, and is of a neuralgic nature. Paroxysmal fever not unfrequently succeeds to such neuralgias, and also to pains in the region of the left ovary. In other cases, the splenalgia is the consequence of a genuine splenitis, that may perhaps have been induced by external violence. Whatever may be the cause of the malady, there is always a very obvious relation between the suffering of the spleen and the paroxysmal fever. Organic alterations of this viscus give rise to such attacks; and these are liable to return as long as the lesion remains uncured. It is only when the spleen is destroyed by new morbid productions, that we find exceptions to the truth of this position.

8. Any disturbance or lesion of the gastro-enteric apparatus must be regarded as an accidental complication, and not as the cause, or as even a direct effect, of intermittent fevers.

9. We have no reason to believe that they (agues) are produced by any alteration in the state of the blood. The marsh-miasm seems to act directly upon the spleen—occasioning in the first place an hypertrophy of its substance, and subsequently the paroxysmal fever—in consequence of some specific influence which we do not understand.

10. Diseases of the urinary passages sometimes give rise to febrile paroxysms, generally of a quotidian character. Many reasons lead me to believe that in such cases, as well as in certain instances of uterine and ovarian disease, a morbid impression is made on the splenic plexus of nerves. In chlorotic girls, in whom the spleen has become enlarged, and who suffer from intercostal neuralgia on the left side, such attacks are of frequent occurrence.

11. If we consider the intermittent and periodic character of neuralgia, in connexion with that of paroxysmal fevers—the manner in which both diseases yield to the sulphate of quinine—the frequent coincidence between certain neuralgiæ and paroxysmal fevers, &c. we shall be led to the conclusion that the 'point de depart' of these fevers is most probably in the nervous system, and especially in that part of it which is distributed to the spleen.

12. Intermittent fever may exist without any hypertrophy of the spleen being present; but then the paroxysms are not well marked. Such cases are rare; and even when they are met with, the attentive physician will generally detect, by means of delicate percussion, some morbid affection of this viscus.

13. Hypertrophy of the spleen may cause ascites and anasarca. The dropsy will quickly disappear, if the visceral disease be cured. A few *grammes* of quinine will often suffice to dissipate the splenic engorgement in the course of 24 hours (!) and the dropsical effusion in three or four days.

14. In a state of health, 50 centigrammes of quinine, whether received into the stomach or injected in an enema, will often cause a sensible diminution in the size of the spleen,(!) analogous to what takes place in hypertrophy of that organ.

15. From a multitude of observations it appears that the sulphate of quinine, in the dose of from one to three *grammes,* has the effect of diminishing in the course of five minutes (dès la cinquieme minute!!) the size of the spleen; and also that the bisulphate, the acetate, and the citrate of quinine determine a sensible diminution in the course of one minute!!! (This certainly beats all medical marvels that we ever read of—in the 19th century too!).

16. In the treatment of paroxysmal fevers, and of hypertrophy of the spleen, the quantity of the preparations of quinine to be administered should be proportioned to the degree of the hypertrophy.

17. In certain erratic fevers, in which—although this is of very rare occurrence—there is no co-existent splenopathy, the quinine may be advantageously administered in small doses.

18. In the present state of medical knowledge, we may fairly assert that the quinine acts, in the cure of paroxysmal fevers, by rectifying the morbid state of the spleen. In persons free of such fevers, but in whom this viscus is enlarged, a sensible diminution of its size is effected by the use of the medicine. We may therefore infer that it exerts its salutary influence not directly on the fever, but on the splenic affection.

19. Quinine is of very decided utility in many cases of remittent and even of typhoid fever, but only in such as are accompanied with enlargement of the spleen.

M. *Piorry* sums up his long string of propositions by ascribing all their merit to the use of *plessimetric percussion;* and thus modestly hints at the greatness of the discovery which he has effected. "The art," says he, "of striking with tact a flat piece of ivory, accurately applied over the organs of the body, has thus enlightened a most important point of practical medicine, I mean the history and treatment of intermittent fevers. It has also served, and will continue to serve, to measure the therapeutic effects of many medicines—so true is it that we should not neglect the use of any means, and that we should ever bear in mind the memorable saying of one of our distinguished countrymen, when speaking of the thermometer:

'When we see such great results obtained from the simple aid of a little mercury inclosed in a glass tube, and when we remember that a small piece of steel, suspended on a pivot, led to the discovery of the New World, it ought surely to teach us that nothing, which can enlarge and exalt the senses of man, should be deemed of little moment; and this motive will plead my excuse for the multiplicity of details into which I have entered.' (Biot, Traite de Physique.)" —*L'Examinateur Medical.*

---

### M. Andral on the Carbonic Acid exhaled during Respiration.

The conclusions, with which this accomplished *physiological*—not in the sense of the Broussaian School—physician sums up a long memoir which he recently read on the above subject, at the Royal Academy of Medicine, are the following:—

1. The quantity of carbonic acid, exhaled from the lungs in a given space of time, varies with the age, the sex, and the constitution of the individual.

2. In the male, as in the female sex, the quantity is found to vary according to the age, independently of the bulk or weight of the person on whom the experiment is made.

3. At every period of life, between the eighth year and the most advanced age, a marked difference is observed in the quantity of the carbonic acid exhaled in the male, from what is found to be exhaled by the female. It is, *cœteris paribus*, always much more considerable in the former than in the latter. This difference is especially remarkable between the 16th and 40th years of age—a period during which the male very generally exhales almost twice as much as the female.

4. In the male, the quantity exhaled goes on steadily increasing from eight to thirty years of age. This increase is greatest at the period of puberty. After the 30th year the proportion exhaled begins to decrease; the amount of decrease becoming more and more considerable in advanced age, so that in the latest period of life, it is nearly about the same as it was in early youth.

5. In the female, the exhalation increases according to the same law as in the male, during the period of childhood. But at the time of puberty, when the menstrual function begins to be developed, the increase—contrary to what occurs in the other sex—is suddenly arrested, and remains stationary (at nearly what it was in infancy) as long as this function continues in its integrity. When it begins to cease, the exhalation of carbonic acid from the lungs increases in a very remarkable degree. As the woman however advances to old age, it again decreases, and ultimately it becomes, as in the male sex, very small.

6. During the whole period of pregnancy, the exhalation is for the time increased to what it is usually in women "parvenues a l'epoque de retour."

7. In both sexes, and at all periods of life, the amount of the exhalation is proportionally greater in persons of a robust constitution and of a muscular frame. Whenever the energies of the system are reduced from disease or otherwise, the quantity of carbonic acid exhaled from the lungs is found to be diminished.

In conclusion, M. *Andral* remarked that the variations described above do not depend, as one might *à priori* imagine, on differences in the mere capacity of the thorax; although, as a matter of course, they are a good deal influenced by this cause.—*Encyclographie des Sciences Medicales.*

---

### Case of Gingival Diptherite, with Remarks.

A woman, 40 years of age and apparently of a healthy constitution, began to experience, without any appreciable cause, a painful and puffy state of the gums. On examining them, they were found to be red and much swollen. At the point of their junction with the teeth, they were covered with a greyish-coloured dirty-looking crust or slime, from beneath which blood oozed out on the slightest pressure of the parts. The breath was very offensive; the submaxillary glands were somewhat swollen; and there was a little feverish *malaise* of the system. This is the disease which M. *Bretonneau* first called "gingival diptherite," and which some of the old authors have described under the names of "scorbutic gangrene of the gums," and "aquatic chancre." We may here remark that it is quite a mistake—which, by-the-bye, still prevails among some medical men—to imagine that the malady has anything to do with genuine scurvy. Indeed, on many occasions, it appears to attack the robust and healthy rather than the feeble; and, moreover, it is seldom or never associated with hæmorrhage from other parts of the body.

If the dirty-looking crust be removed from any part, the mucous membrane underneath will be found eroded, or even ulcerated, and the gums red and seemingly inflamed; but not of a purplish colour, as in scurvy. If muriatic acid or alum be applied, this covering or crust becomes detached; but it is quickly re-secreted.

The disease may continue stationary in this simple state for weeks, or even months; but in many cases it spreads to the internal surface of the cheeks, which then becomes the seat of the fibrinous (*couenneuse*) secretion, and this adds much to the fætor of the breath.

The *diptheritic* inflammation may even extend back to the uvula and tonsils. When this takes place, symptoms of malignant cynanche make their appearance; and if the disease be not speedily arrested, the larynx itself becomes affected, and the patient will inevitably die. It is therefore of great importance to form at first a correct diagnosis of the disease; and here we must not omit to mention a curious circumstance which may not be known to many medical men. True diptheritic inflammation rarely extends from the gums to the larynx; but it is by no means unfrequent that the laryngeal disease is caught by the attendants who wait upon the patients labouring under the gingival affection.

M. *Bretonneau*, many years ago, directed the attention of the profession to the circumstance that, during the prevalence of epidemic cynanche maligna, many persons were affected with 'couenneuse' inflammation of the surface. The truth of this observation I had many opportunities of observing during the great epidemic of Diptherite which prevailed in many districts of France in 1828. The surface of wounds and ulcers was apt to become covered with a fibrinous exsudation or coating, which in many instances continued unchanged for weeks and even months. At the same time, croup was very prevalent among children, and gingival diptherite was not unfrequent in adults.

Those, who have read the work of M. *Bretonneau*, will remember that the epidemic of croup, which committed such ravages in Tours, was supposed to have been conveyed thither by a troop of soldiers who were found on their arrival to be affected with gingival diptherite.

In confirmation of the views now mentioned, M. *Trousseau* (the writer of the preceding remarks) informs us that, when his patient was admitted into the ward, he expressed his fears that her infant would become affected with croup—although it was perfectly well at the time. The prediction proved unfortunately to be too true; for the child soon after died from the affection of the larynx.

The treatment of gingival diptherite is sufficiently simple. The affected parts should be touched every morning with strong muriatic acid; afterwards once every second morning will be often enough. During the day, a mixture of borax and honey should be frequently used as a gargle.—*Gazette des Hôpitaux.*

---

### Danger of excessive Depletion in Pericarditis.

On the occasion of a case of membranous pericarditis, accompanied with effusion, which was recently treated in the medical wards of the Hôtel Dieu, M. *Tessier* the physician made the following practical remarks.

This phlegmasia of the pericardium is to be regarded rather as one of the forms of rheumatism than as a mere localisation of this disease. When the inflammation is attended with an effusion of membranous lymph on the surface of the heart, the prognosis must always be considered unfavourable. The case then very frequently terminates in a fatal manner, either rapidly or by a more slow and chronic process. When a fatal termination is likely to take place, there is very generally a sharp pungent pain felt in the cardiac region; and this is usually accompanied with great embarrassment in the movements of the heart, with syncope, breathlessness, and sense of impending dissolution. These symptoms become more and more alarming, as the amount of the serous effusion within the bag of the pericardium increases.

The treatment of such complaints is often most perplexing; as the practitioner is apt, in particular cases and at a certain stage of the disease, to attribute some of the symptoms to an aggravation of the inflammatory mischief, when in truth they are owing to an anemic state of the constitution (induced by the evacuations that have been rendered necessary), and when therefore they require not the continuance of depletory, but the administration of strengthening *remedies*. I remember well a case in which my experienced colleague, M. *Recamier*, diagnosticated (the word, though most cacophonous, is expressive) such a condition of the system in a patient who exhibited all the symptoms of genuine pericarditis. The pulse was 180; but the pulsations were rather mere vibratory movements than distinct appreciable beats. (Surely with such a state of pulse, no evacuations, far less the direct loss of blood, can ever be proper.—*Rev.*) The medical men in attendance wrongly supposed, from the great frequency of the pulse, that the use of antiphlogistic measures was still required. The patient indeed always seemed to be relieved for a time by the abstraction of blood; but ere long the symptoms returned, and with aggravated severity.

M. *Recamier* being called into consultation at this period of the case, and finding on auscultation that both sounds of the heart were very *clear*—a useful diagnostic symptom—concluded that the symptoms were the result not of any *recrudescence* of the inflammation, but of the actual want of blood of the vessels, or of a veritable anemia. He advised the immediate discontinuance of all evacuating, and the use of strengthening medicines; from that time the patient began to recover.

M. *Tessier* alludes to a similar case which recently occurred in his own practice, and which he saw along with another physician. All the symptoms of pericarditis with effusion were present; the pulse was very rapid and extremely irregular. The use of antiphlogistic remedies had removed the pain and all the active symptoms; but still the distressing cause of anxiety and the unnatural state of the circulation continued. The sounds of the heart on auscultation were unusually sharp and *clear*. After the cautious administration of wine, the rapidity of the pulse subsided, and the inward distress became less and less: sinapisms were applied over the region of the heart. The patient did well.—*Gazette des Hôpitaux.*

*Remarks.*—Nothing to our minds is a more convincing proof of the very *unpractical* character of the French school, than the not unfrequent announcement of old-established and well-recognised (at least in this country) medical truths, as if they were discoveries of the present day. And although M. *Tessier*, in the preceding remarks, may not lay claim to any originality as to the precepts which he lays down for the management of certain cases of pericarditis, the whole turn of his lecture evidently gives the impression to the reader that the value of these very precepts is, in some degree, novel to himself. Most of the French physicians have for so many years allowed themselves to be so thoroughly imbued with the exclusive doctrines of the Broussaian school, that they have scarcely been able to see more than one, and the same, morbid element at work in whatever diseases came under their inspection. From the plague and the Asiatic cholera to a simple 'embarras gastrique,' or an old man's Winter cough, all alike have been regarded as only modifications or degrees of an inflammatory affection; and, consequently, almost every case has been subjected to one method of treatment.

Had such a doctrine prevailed on this, instead of the other, side of the Channel, it might have given rise to less surprise, seeing that the diseases of the English people are on the whole of a more inflammatory character and will stand the use of depletion better than those of their more abstemious and thin-diet neighbours. But fortunately for John Bull, his doctors are not very much addicted to theorising, and are usually in the habit rather of looking at things as they present themselves before their eyes, than of speculating about their cause and origin, or determining beforehand in what manner they are to be treated. Not so with the French physicians generally; they are taught in the schools to regard diseases as certain uniform entities, which exhibit the same, or at least similar, characters in all individuals and at all times, and which may therefore be arranged with almost as great precision and certainty as plants are in a botanical, or animals in a zoological, catalogue—a great and a very pernicious mistake.

But not to be led into any vague generalities, let us revert for a moment to the subject of excessive or over-prolonged depletion in the treatment of inflammatory diseases.

Whenever the pulse becomes exceedingly hurried, irregular and compressible, the physician may rest assured that the period for ceasing further evacuations has arrived, and that the use of mild tonics, and perhaps of sedatives also, is now required. The mere existence of pain, or of difficulty of breathing, or of cough, or of sickness, or of delirium, or, indeed, of any one symptom, is not to be regarded, *per se*, as a sufficient indication for the use or continuance of antiphlogistic means; for we well know that each and all of them may depend upon an *ancæmic*, as well as upon a *plethoric*, state of the system. A dose of opium, given at the right moment under such circumstances, will often act as a charm by sending Nature asleep, and thus lulling, for a time, the irritable state of the nervous and circulatory systems. If this advantage be followed up by a judicious use of tonics and mild food, many a case will be quickly conducted to a favourable termination; whereas, under a different management, weeks and months would have been fruitlessly spent in endeavouring to restore the patient.—*Rev.*

---

### M. Recamier on Critical Days in Disease.

The following remarks of this truly eminent and most practical physician deserve notice, from their savouring not a little of the doctrines of the last century. Is it that, in medical as in theological matters, there is a tendency in the present day to recur to ancient usages and opinions?

"The proper period for interfering in diseases cannot be determined beforehand, or by any fixed and uniform rules. This moment varies in different cases of the same disease, in consequence of a variety of circumstances arising from peculiarities either of constitution, age, sex, and so forth. Do we find, that all young people arrive at puberty at the same age? or is the process of digestion completed within the same period in all persons alike? Certainly not; one person digests, in the course of two hours, food which may require four or even six hours in another person. And so it is with diseases; they are subjected to a sort of digestion, the duration of which is found to vary not a little in different constitutions. The *coction*—in other words, the crisis, whether this occurs by perspiration, by the urine, by suppuration, or in any other way—is usually effected in a certain number of days: this we may calculate upon in many cases to a certain degree; but the period may be influenced by many unforeseen circumstances. The physician cannot be responsible for accidents; these may be regarded as episodes occurring unexpectedly in the course of a disease, and perhaps will wholly change the results that might previously be considered highly probable.

“ But it is not so much upon the number of days that may have elapsed since the beginning of a disease—although this consideration is often of the highest importance—as on the actual state of a patient when we first visit him, that our prognosis on the one hand, and the therapeutic indications on the other, are to be based. I have bled pneumonic patients on the fifteenth and eighteenth days after the attack, when I have found the pulse remaining hard, and provided there were no symptoms of suppurative action having already commenced.

“ It is, therefore, the actual state, rather than the length or duration, of a disease that we are to attend to. This latter circumstance, indeed, is not to be neglected in our calculations; but it must not be trusted to as a guide, since the *coction* or the degeneration of a disease may not occur on the day that we expect it to take place.

“ You may have heard, that much stress has been laid by some writers on the ninth day, as the most frequent period at which the crisis of pneumonia is apt to take place. What I have just now said, as to the necessity of subordinating the consideration of days to that of the crudity or coction of the disease, and to the particular indications deducible from the state of the pulse or the general strength of the patient, must not make you overlook the real value of *days*, especially in reference to the occurrence of crises, and to the formation of our prognosis. For example, any disease, which has lasted longer than one day, will in all probability continue for half-a-week at least; and if it exceeds this period, it will certainly reach the beginning of another week. If the crisis occurs on the seventh day, we may be certain that it will not pass beyond the ninth; but if it does, we may then count upon it continuing for three weeks. As to the seventh day, there is, in general, very little to be done; the crisis may occur on the following two days; if it has not commenced on the seventh, it may take place on the ninth; and if not on the ninth, we must wait till the 11th. When it exceeds this period, it becomes very difficult to say when it will occur; the disease may be prolonged to the 21st, the 28th, or the 30th day. I have occasionally seen it deferred till even the 49th day.

“ We must never allow the consideration of particular days to influence our treatment of pneumonia; as long as there is much excitement present and the pulse remains hard and full, blood must be drawn; but, whenever it becomes soft and compressible, we should cease to bleed.

“ The following case will show well how necessary it is to be guided by symptoms as they arise in the treatment to be pursued. A man was admitted into the hospital with the usual symptoms of jaundice; his pulse was soft, and there was but little or no febrile re-action. Laxatives and emollient remedies were tried for several days; but still there was no appearance of resolution. It was now found that the region of the liver was slightly painful, when pressed on: a few leeches were therefore applied over the tender spot. Almost immediately afterwards, there was a marked aggravation of the febrile symptoms, and much more re-action on the system. The patient was now bled from the arm; there was no relief;—the bleeding was therefore repeated; but still without removing the tenderness—and as the blood drawn was always buffy, and the pulse had risen in force after every depletion, the venesection required to be repeated other six times, before the inflammatory symptoms freely yielded. After the eighth bleeding, a crisis by perspiration and copious purgation ensued, and the patient was speedily relieved from all his sufferings.

“ I cite this instance,” says M. *Recamier*, “ to show how necessary it is to watch the state of the pulse in all cases, as strongly marked inflammatory symptoms may unexpectedly occur in the course of a disease, which in its first stage was attended with symptoms of debility and want of power. It affords a very instructive illustration of the difference between mere *oppression*, and a real *abolition* or loss, of vital force."—*Gazette des Hôpitaux.*

---

### PNEUMONIA ACCOMPANIED WITH DELIRIUM: USE OF ANTISPASMODICS: REMARKS.

A man was admitted into the physician's wards at the Hôtel Dieu with the ordinary symptoms of pneumonia, when passing from the first to the second stage (crepitant râle throughout the entire extent of the lung). Next day, the respiratory *bruit* was much more obscure: and on the following day it could scarcely be heard at all. From this period, a distinct blowing sound was perceptible under the axilla; the sputa exhibited the appearances usually observed in pneumonia. The patient was immediately bled very freely, and also cupped. In the evening, he became delirious; but there was scarcely any febrile excitement of the system: the delirium continued throughout the night. The patient was ordered repeated doses of syrup of poppies, and also a grain of musk in the form of pill twice a day. For the first day or two, there was no decided amendment; but the dose of the medicines being then increased, the delirium began to subside, and by the eighth day it had entirely ceased; and with it the pneumonia at the same time. It was very curious, throughout the progress of this case, to observe the subsidence of the thoracic, simultaneously with that of the cephalic, affection.

The following remarks were made by M. *Tessier* on this subject.

"Every one knows full well that when pneumonia is accompanied with delirium, the prognosis is necessarily very unfavourable, if the latter symptom be not quickly subdued: the complication is usually the more serious, when the delirium is unattended with fever. On the other hand, it is remarkable that the pneumonia usually yields with great readiness, when the head disorder gives way to the remedies employed. The latter is therefore, under such circumstances, to be regarded as the more important indication of the two, in respect of the treatment to be adopted; experience having clearly shewn on very numerous occasions, that by removing the delirium, the pneumonia will subside of itself; whereas, if the delirium continues or does not yield to the remedies used, the inflammation will inevitably prove fatal."

In allusion to another case of pneumonia in the hospital, in which there was exceedingly little re-action in the system, and which yielded to blistering and the use of the Kermes mineral internally, without recourse being had to venæsection, M. *Tessier* very properly insisted on the necessity of being guided by existing symptoms in all cases of ambiguity, and of not being led away by any preconceived notions as to the intrinsic nature of the disease.

"Inflammation of the lungs is not to be treated in all cases alike by the same means; and those, who attempt to establish a particular method of treatment by appealing to statistical tables of cases and so forth, commit a very egregious error. To talk therefore of what should be done at the commencement, and what at the middle, and what in the advanced stage, of a disease, without having regard to the existing symptoms, manifestly argues a sad want of practical knowledge. We must be guided in our treatment by the symptoms present, and not by the circumstance of the disease having existed this number, or that number, of days. Whether it is the fourth, or the sixth, or the tenth day, of the attack, on which we bleed, it matters nothing—provided the symptoms require that depletion should be practised: it is not a question of time; it is a question of indication. If, for example, in the present case, I had paid regard to the statistical precept that bleeding is always much more useful at the commencement than in the more advanced stage of pneumonia, I should have committed a very grave mistake; for here there was no indication to bleed at any time; the pulse was weak and compressible, and the patient was in such a state that in all probability a loss of blood would have brought on syncope and general prostration, that might have terminated fatally."—*Ibid.*

---

### On Animalculæ in the Blood.

The occasional existence of worms in the blood of a living animal is a phenomenon that must excite the curiosity of every one. The allusions to such an occurrence in the writings of the old physicians are so imperfect and confused, that it is difficult to determine what amount of credit we can attach to them. If the observations of Welsch* and Polisius† on worms found within the heart of the human subject are not to be received as authentic, these authors having probably mistaken fibrinous concretions of the blood for actual worms, recent researches however have clearly shewn that such entozoa do really sometimes exist in the hearts of some of the lower animals. M. Barkow‡ found in the right ventricle of a species of heron (*Ardea Cinerea*) two nematoids which are now preserved in the museum at Greifswald; and M. Baer§ has discovered a peculiar species of the distoma (*d. duplicatum*) in the hearts of several Molluscous animals.

It is well known that a particular kind of worm (*Strongylus armatus minor*) is occasionally developed in the fibrinous concretions that form in aneurismatic arteries of Solipedous animals. The veins too of certain animals are not exempt from these entozoa. Treutler|| found a species of *fasciola* in the pulmonary veins of a seal: and another kind of worm in the vena cava of a deer. Numerous authors have subsequently studied the particular species of *Strongyli*, that are occasionally met with in the venous sinuses at the base of the cranium and also in the pulmonary veins of the *Delphinus Phocæna*. Schmitz, while observing the circulation in the mesentery of a frog, happened to observe the appearance of living animalcules existing in the blood. To ascertain if this was of frequent occurrence, he examined the blood of 53 lizards, and of 81 frogs; but without success. At length however, in a frog, he again discovered in the blood animalcules similar to those which he had seen in the first instance. He observed that they made their way through the parietes of the vessels, and then burst and discharged the granular matter which their bodies appeared to contain—a phenomenon that has been repeatedly witnessed in certain *Polygastric* animalcules, &c. It is the opinion of M. Valentin that these blood entozoa, described by Schmitz, most nearly resemble the *Polystoma venarum* of which Treutler has given so minute an account.

M. Valentin himself has repeatedly found in the blood-vessels of frogs the *Anguilula intestinalis*. His idea is, that these animalcules are hurried along in the torrent of the circulation, till they, as it were instinctively, stop in some organ that is best suited as a domicile for them; and that they then work their way through the parietes of the vessels. This author also mentions that a *Strongylus armatus* was recently discovered in the blood from the venæ cavæ of a horse; there was no trace of an opening in the walls of the vessel, or of any communication between it and the intestinal canal.

---

* Disput. de Verme Cordis. Lips. 1694.

† Observ. de verm: in corde repert: (Ephem. Nat. Cur.)

‡ Observationes de Entozois. 1825.

§ Novæ Obs. de Entozois. 1829.

|| Observ. Anatomicæ-Pathol: de Helminthologiâ. 1793.

Dr. Vogt, while examining with a microscope the membrana nictitans of a frog, was surprised to find that the vessels, still filled with blood, contained numerous living animalcules, which moved about with great vivacity: similar animalcules were observed in the blood-vessels of other parts of the body. On inspecting the abdominal cavity of the animal, he observed several brown-coloured saccules, similar to those in which M. Valentin has occasionally met with *filariæ*. As he believed that these minute worms are the embryos of entozoa, he examined these saccules with great attention; but they were all quite empty.

In other frogs, however, in which these abdominal saccules existed, he found that many of them contained a *filaria*—the resemblance between which and the animalcules of the blood is very marked.

Valentin has recently described, in a letter addressed to Müller, some very curious phenomena, which he observed in the blood taken from the abdominal aorta of a trout. On first examining it, he noticed a number of dark-coloured globules mixed with the globules of the blood; they were in rapid movement, without however changing their place. After the lapse of a short time, I was surprised, says he, to perceive first on the side of these globules a sort of transparent appendage or tail; and subsequently that there was evolved an animalcule of an elongated shape, and which was continually in rapid motion round its own axis. This animalcule belongs probably to the old genus *Proteus*, or what Ehrenberg has called *Amœba*. Its length did not exceed from a 3 to a 5,000th part of an inch. Sometimes as many as ten and even more could be counted in a single drop of the blood; but in no other part of the body could they be discovered. The only other entozoa that were found were several ascarides in the pyloric appendages.

M. Gluge of Brussels has subsequently communicated to Müller's Archives a description of an animalcule, which seems to be very similar to those mentioned in the preceding notice, and which he accidentally met with in the blood of a frog.

It seems therefore that the existence of entozoa in the blood of certain animals is by no means of very rare occurrence. As yet we know nothing of the manner in which they become developed; but further investigation of the subject may throw light on this very curious subject.—*Archives de Medecine Comparée,** No. 1. Oct. 1842.

---

### NOTE ON EMPHYSEMA OF THE LUNGS—CASE OF THE LATE MR. HORNER—GREAT SAGACITY OF DR. BAILLIE.

A few pages back we have given a brief account of a discussion at the French Royal Academy of Medicine on the subject of Pulmonary Emphysema. Since writing that article, we have been reading the recently published memoirs of that distinguished statesman and most amiable man, the late *Francis Horner*, M.P.; and his case seems to us to be altogether so very interesting in a medical point of view, and so well calculated to throw some light—as far as a single example can do—on the history of the disease in question, that we do not hesitate to give the details at some length, as recorded in the work alluded to. Our readers will observe that, in many respects, it bears out the opinions expressed by M. *Louis*

---

* (This is the first number of a new Journal recently started in Paris under the immediate management of M. *Rayer*. It promises to be of great interest; the study of comparative pathology has hitherto been far too much neglected.—*Rev.*)

and M. *Bouillaud*, &c. on the frequent co-existence of Cardiac affection with Emphysema of the lungs.

Mr. *Horner* seems never to have been a very robust man; his constitution or temperament was of the bilious-phlegmatic kind; and for many years he had been subject to stomach complaints. In 1816, the year preceding his death, he began to exhibit symptoms of pulmonary disease, the nature of which could not be very easily made out. In the Summer of that year we find him writing thus to his father: "I am still a little plagued with a cough, in which there is nothing at all material, except the circumstance of its continuing so long, which I think is owing to the cold weather. To be sure of this, I have seen Dr. *Warren*, who thinks there is nothing in it; but considers the stomach, as of old, chiefly in fault, and has given me some directions to observe on that head." We gather from a subsequent letter that, along with cough, difficulty of breathing, amounting often to breathlessness, and palpitations of the heart, there was extreme muscular debility; for we find him at one time alluding to a "feeling of mental lassitude that seemed to wither me within," which he had experienced at Edinburgh in the course of the Autumn.

The opinion of his physicians both in that city and also in London was, that his complaint was some affection of the lungs, neither phthisical, nor yet dropsical; but they felt considerable embarrassment in determining its real nature. The advice of all was, that the Winter should be passed in a warm climate, and that all active exercise, including much talking, should be avoided: "No vociferation Sir, even if you are paid for it," was the injunction of Dr. *Gregory*. He went to Pisa; but the change does not seem to have produced any very decided benefit, for we find a friend writing to him in the first week of January thus: "As your breathlessness seemed not to be at all relieved either by the change of climate, or by the treatment recommended to you by *Baillie* and *Warren*, I made out a statement of your case at present, as well as I could collect the particulars from your own letters and your brother's, and sent copies of it yesterday to both these physicians, with a request that they would take it into their consideration, and give me their opinion...... *Baillie* thinks it (your illness) may proceed from a consolidation of part of the substance of the lungs, in consequence of which there is less space for air, or it may arise from a change of structure in the air-cells, by which they are become larger, and in the same proportion afford a smaller surface for the oxygenation of the blood. In either of the last suppositions there is no danger from the complaint, though there may be much inconvenience. They recommend to you to resume the use of the mercurial pill, and to try the effect of the supercarbonate of potash."

While at Pisa, Mr. *Horner* put himself under the care of Dr. *Vacca Berlinghieri*, who seems also to have been much puzzled about the case. Opium, in doses of a grain at bed time, (and this was occasionally repeated in the morning), was found to procure great relief to the dyspnœa: "The relief," he writes himself, "seems to me quite marvellous, and I could fall down and worship my pill like a Turk; what is very new to me indeed, I have got through the labours of my toilet, not only without pain and palpitations, but with scarcely any feeling of exertion; and I am altogether a stronger and a better man than I have been a great while." From the very decided relief which the use of the opium seems to have invariably given, Dr. *Vacca* thought it reasonable to believe that the chest symptoms were, in part at least, owing to an affection of the nerves of the lungs.

As usual with most patients labouring under thoracic complaints, Mr. *Horner* appears never to have entertained any apprehension himself about the result of his malady: there is usually an innate hopefulness not only in phthisis but in most pulmonary complaints that is truly surprising. The fatal change at last seems to have been very sudden and unexpected; the dyspnœa and cough became worse, and there were strong palpitations of the heart, with a low, irregular, and intermittent pulse, and general prostration. His brother had left him for a few minutes, and on returning to his bed-side found his face deadly pale, his eyes fixed, and his hands cold: it was thought at first that he had merely fainted; but it was soon found that life was extinct.

The following report of the appearances found on dissection was drawn up by Dr. *Vacca.*

*Sectio Cadaveris.*—The body was not much emaciated, All the abdominal viscera were perfectly sound; only the venous system was unusually gorged with blood. On opening the thorax the lungs were observed to be singularly shrunk, especially the right one. Their colour was livid, and their surface was very uneven: this unevenness was produced by a great number of transparent vesicles, varying in size from that of a pea to that of an almond. By far the greater number of these vesicles were on the anterior face of the lungs; few on the posterior. On compressing them they disappeared, and the air, with which they were filled, passed into the bronchi: they re-appeared on blowing air into the trachea. These vesicles did not communicate with the cellular tissue, which unites the air-cells together, so that the case must be regarded not as one of Emphysema, but of an abnormal dilatation of the air-cells. A great portion of the substance of the lungs, especially at their posterior part, was condensed, indurated, and in many places completely hepatised. The different lobes did not adhere together; neither was there any adhesion between the pulmonic and the costal pleuræ. The lymphatic glands of the bronchi were larger than usual; and the membrane of the air tubes was slightly engorged.

The pericardium was sound; it contained a small quantity of serosity. The heart was extremely soft and flaccid, so that it was readily torn with the fingers. Its right auricle was much dilated, and filled with blood. The walls of the corresponding ventricle were much attenuated; it was in them that the flaccidity of the muscular substance was most conspicuous. Within its cavity was a firm, fibrous, and whitish-coloured coagulum, which adhered very strongly to the *columnæ carneæ.* This coagulum had been most probably formed during the last moments of life. The right cavities of the heart did not exhibit anything unusual.

*Baillie,* in his Morbid Anatomy, and *Lieutaud,* in his Historia Anatomico-Medica, have related several cases of morbid change which have some resemblance with the present one; but I do not observe that either of these authors had ever found shrinking of the substance of the lungs, dilatation of part of the air-cells, hepatisation of a large portion of the pulmonic parenchyma, and an affection of the heart, in the same individual.

Docteur Vacca Berlinghieri.

Pisa 12th Fevrier, 1817.

The following note from the late Dr. *Warren* (for many years one of the leading physicians in this Metropolis), in reference to the preceding report, will be also read with interest.

"I have shewn *Vacca's* account to Dr. *Baillie,* who considers the case as exhibiting a very unusual form of disease, and one which is evidently out of the reach of medicine. The state of the heart presented no unusual appearances; the flaccidity and tender structure of its fibres being met with very frequently in individuals whose constitutional powers have failed by slow decay: the appearance within the right ventricle was a coagulum of blood not uncommonly found in that situation after death. The condensation of the lungs is also not unfrequently met with, and justifies the opinion which Dr. *Baillie* held to you of such an alteration of structure being the probable cause of Mr. Horner's difficulty of breathing—which was never attributed to water in the chest, but to an obstruction in the circulation of the blood through the lungs, arising from some cause not easily distinguishable. The enlargement of the air-cells to the extent mentioned by Dr. *Vacca* is a disorder so rare, that there are only three instances to be found in the anatomical collections with which Dr. *Baillie* is acquainted. The immediate cause of death appears to have been owing to the increase of the obstruction of the lungs to such an extent, as to have prevented the free passage of the blood through the branches of the pulmonary artery, by which the right side of the heart become gradually gorged with blood, and its action was slowly suspended.

PELHAM WARREN."

*Remarks.*—This case is altogether an exceedingly interesting one, and forms a good theme for a little practical discussion. What was the primary complaint,—the 'fons et origo,' of the long-continued suffering, and finally of death? It was said at the time, and this too by men of the highest professional authority, that it was Emphysema or morbid dilatation of the air-cells with partial solidification of the substance of the lungs, and it was regarded, as we have seen from the preceding report, as an instance of an extremely rare form of disease. But it may be very fairly asked, was the pulmonary complaint the primary, or was it not rather the secondary, affection? Was it not the result of the dilated atonic condition of the heart? We think it was; and that the case of Mr. *Horner* was in fact one rather of cardiac, than of pulmonic, disease. From the want of energy in the ventricles, more especially in the right one, they were unable to propel with ease the stream of blood that was continually pouring in. The necessary consequence of this was, that the circulation through the lungs was more and more embarrassed, until a complete congestion took place in certain parts, and the pulmonary parenchyma thus became impervious to the air. The remaining portion of the lungs had to compensate for the deficiency, and had therefore extra duty to perform: hence the dilatation of the air-cells, and hence the dyspnœa and also the distress in the action of the heart. The history of the case seems to confirm this view of its pathology: the symptoms were always much aggravated by bodily exertion, and they were remarkably relieved by the use of opium—which no doubt tranquillised the movements of the heart for a time, and thus enabled it to discharge its contents more completely and more regularly.

Does change of climate hold out any prospect of benefit in such cases?—we think not. Rest and quietude in a pure atmosphere, passive exercise in a carriage when the weather is fine, great attention to the stomach and bowels, the use of mild tonics and antispasmodics, perhaps smoking, and certainly cheerful occupation of the mind—these are our chief remedies in such a case. To these we might be inclined to add, in most instances, a seton over the region of the heart. The grand thing is to form a correct diagnosis; the proper treatment follows almost as a matter of course.—*Rev.*

---

### MISCELLANEOUS NOTICES.

### 1. *Acupuncture in Neuralgia.*

M. *Lallemand* of Montpellier has for many years been in the habit of using acupuncture in cases of *genuine* neuralgia with very decided benefit: against rheumatic pains, he says, it is quite inefficacious. We must therefore be careful to discriminate the cases for its employment; otherwise we shall certainly be disappointed. If the pain be limited to the *trajet* of the nerves, we may with tolerable confidence promise relief, if not a complete cure, of the suffering. M. *Lallemand* relates many cases: one we shall briefly notice. A man had for six months been afflicted with most severe pain along the whole course of the sciatic nerve; five needles were inserted along its tract, and left in for three hours. The application was repeated, at intervals of one or two days, four successive times; and the man was then completely cured.

2. *Importance of Veterinary Medicine.*

At the recent annual meeting at Strasbourg of the Scientific Association of France, M. *Falk* read a very sensible paper with the view of shewing the importance of medical men making themselves acquainted with Veterinary medicine. The utter neglect of this study is certainly to be regretted, as some valuable hints for the treatment of diseases in the human subject might be derived from what may be called Comparative Pathology. Every one in the present day recognises the importance of a knowledge of the anatomy of the lower animals;—why not then of their diseases also?

The suggestion of M. *Falk* was well received by the Association.

3. *French Gratitude to Medical Men.*

We observe by one of the Paris Journals that the names of *Percy, Desgenettes,* and *Larrey* have been recently engraved on the famous Arc de Triomphe, at the Barriere de l'Etoile.

In some respects certainly, medical men occupy a higher position in society in France than in this country. For example, who ever heard of a doctor becoming a *Baron,* or peer of the realm with us? and yet every one knows that *Portal, Dupuytren,* and *Cuvier* occupied that dignified rank in the parliament of France; and where is the nobleman of Germany that might not be proud to be associated with a Baron *Humboldt*? True, the circumstance of a French peerage being merely personal, and not hereditary, makes a very material difference in the case; but then, why should there not be a certain number at least—we are no friends to an elective aristocracy, as a general question—of the most eminent men in the various departments of science being admitted to the highest honours of the state, without necessarily entailing upon their families the expensive title which they bore themselves?

By a recent Royal Ordonnance, M. *Louis* has been made an officer of the Legion of Honour, and M. *Leuret* a chevalier of the same Order.

Again, MM. *Andral* and *Rayer*—certainly two of the most distinguished names in French medicine of the present age—have been elected members of the Institute. "The selection of such men," says one of the Paris Journals, "shews very emphatically that this illustrious body looks as much to the scientific character as to the practical eminence of its candidates; in a word, that it wants not so much the mere skilful physician as the enlightened philosopher in its ranks."

4. *Treatment of Gonorrhœa.*

"There are two opposite methods of treatment," says M. *Vidal* (de Cassis), "which I deem equally injurious; that which precipitates the issue (qui brusque le denouement) of the case, on the one hand, and that which awaits it with an almost complete inaction on the other. Both of these methods are apt to favour the extension, the displacement, or the chronicity of the disease. It is certainly remarkable that, in almost all the cases of nephritis supervening upon gonorrhœa which have occurred in my experience, the patients have taken the *potion of Chopart,* or some other resinous stimulating diuretic, more or less freely, from the commencement of the attack. Inflammation of the neck of the bladder is not unfrequent after such treatment. The same result is apt to occur when little or nothing is done to check the urethral discharge. The proper line of treatment, therefore, seems to be neither to try to stop the discharge suddenly, nor allow it to take its own course, and leave it to Nature's efforts to remove." (This remark is perfectly just; much mischief might be avoided by acting on M. Vidal's precept.)

5. *Anecdote of Decandolle.*

One day *La Place* being with M. *Cretet,* then minister of the interior, expressed his regret to see *Decandolle,* the ornament of French Botany, sent as professor to Montpelier, as it was the intention of the Institute to have appointed him one of their members. "Your Institute! your Institute!" exclaimed the minister. "I often wish that I could send a cannon-ball among you, and scatter the members over the whole of France. Is it not a lamentable thing to see all the light concentrated in Paris, and the Provinces in utter ignorance?"

### 6. *Poverty of Medical Men: how to eke out a livelihood.*

"One of the most certain signs, (says a writer in the French Medical Gazette), of the 'decadence' of a profession, is the necessity which many of its members experience, to seek for supplementary resources. Medicine is a most noble profession, but in the present day it is certainly one of the worst trades (industries) going. It is all very well to talk of dignity and high feelings; let it be remembered that a doctor has a stomach as well as a heart to attend to. In Paris alone, there are not fewer than 2,000 medical men, and it may be readily supposed that a great number of them scarcely ever get a fee. How then are they to live, until practice comes?—by turning their talents, whatever these may be, to the best account. The other day, I called on a confrere, whose circumstances I knew to be in not a very flourishing condition; I found him busily engaged in painting a portrait; he said that he had sold several of late. But what is your object? said I—'renforcer le metier,' was his answer. If a patient called, he put down his palette, doffed his blouse, slipped on his black coat, and with a grave and becomingly professional countenance went to his consultation-room; no sooner was the interview over, than he returned to his studio, resumed his working dress, and set to his painting again with all the cheerfulness of a brave heart. 'I could not help saying, Apollo is god of the arts as well as of medicine.'

"Several young medical men have exerted themselves with success in the way of 'inventions industrielles;' for example, in the manufacture of portable liquid gas, in the construction of a night-telegraph, in preserving timber for ship-buiding, &c.

"The lamp C———, we owe to the ingenuity of an intelligent physician, who is practising in the suburbs, and who thus diffuses both light and health around him.

"Not a few imitate the example of *Boerhaave, Pinel,* and others, and give instructions in languages, mathematics, &c. translating, perhaps, Virgil and Horace in the morning to one set of pupils, and Hippocrates and Celsus in the evening to another. Often the wife, too, lends a helping hand 'à renforcer le metier,' either in the way of teaching, letting lodgings, or perhaps keeping a little magazine of linen, or silk goods, &c.—whatever, in short, brings grist to the mill!"

### 7. *Compliment to English Genius.*

In a clever defence of the Montpelier School of Medicine from the reproach of having been always too metaphysical in its doctrines, a writer in the French Medical Gazette, says—"The professors, in their lectures, not unfrequently allude to the elementary principles of sound reasoning or logic, and endeavour to test the truth of any novel questions in medical science by appealing to them, on the ground that general philosophy embraces, properly speaking, a code or rule to guide us in deciding upon all matters in detail. Thus it is that our pupils will be found to study the works of the great metaphysicians in conjunction with those of the most celebrated observers of facts, such as *Bacon* and *Hippocrates, Locke* and *Sydenham.*" Of these four names, England claims no fewer than three.

8. *Quinine in Rheumatism.*

Several of the hospital physicians in Paris have been of late experimenting on the effects of quinine in rheumatism, in consequence of the recent recommendation of this remedy by M. *Briquet.* His colleague at the St. Louis Hospital, M. *Devergie,* has published the reports of a good many cases of the disease, acute as well as chronic, in which he administered quinine with decidedly good effects. In some of those where the inflammatory symptoms ran high, bleeding and other antiphlogistic measures were used at first; but in most the remedy was given from the commencement of the seizure. The dose to adult patients varied from fifteen to thirty grains per day. At the close of his communication, he says:—"In my opinion, too much importance cannot be attached to this discovery of M. *Briquet.*" Discovery, indeed!—was not the use of bark in rheumatism strongly advocated by Drs. *Fordyce* and *Haygarth,* more than sixty years ago? and is not mention made of the practice in every English treatise on Practice of Physic? But this only *en passant.*

Neither bark nor its alkaloid salt will ever become a general remedy in rheumatism of the acute kind, except towards the decline of the attack, when the inflammatory symptoms have been subdued, and the pains have become more or less of a neuralgic kind. In chronic rheumatism, especially where the system is feeble and torpid, the use of the decoctum cinchonæ, or of a solution of quinine, is often of very great benefit, either alone or in conjunction with diaphoretic and sedative remedies.

9. *Anti-Neuralgic Pills.*

Dr. *Eisenmann* of Munich, in a well written paper on the general employment of alterative medicines, points out the utility of combining two or more of them together in certain cases of disease. He dwells particularly on some cases of severe neuralgia, which are more or less connected with an agueish state of the system, but which nevertheless resist the effects of bark alone. If however, he says, a medicine, which acts on the nervous system, be combined with the bark, we shall often succeed in effecting a cure. He strongly recommends a combination of quinine, strychnine, and extract of belladonna. [The remark is very rational and just.]

10. *Usual Course of Rheumatism in the Horse.*

M. *Tessier* remarks that M. *Boullay,* one of the most experienced veterinary surgeons in Paris, assures him that the ordinary course of rheumatic inflammation in the horse is the very reverse of what is usually the case in the human subject. In the latter, as all know, the affection of the joints is primary, and that of the pleura, pericardium, or other internal part is consecutive or secondary; whereas, in the former, pleuritis is generally the primary, and the arthritis the secondary affection.

11. *Successful Case of Cæsarian Operation, successful both for mother and child.*

The report of this case is contained in the number of the Journal de Medecine de Lyon, for last February. The woman was 35 years of age, exceedingly deformed from rickets, and pregnant with her second child; the first had been extracted by embryotomy. The necessary incisions were made, and the child, and afterwards the placenta, extracted in the course of two or three minutes. The child cried lustily when taken out, and continued to thrive. The mother was threatened with peritonitis; but this fortunately subsided, and the wound was nearly quite healed by the fortieth day after the operation.

